# Combination of fMRI and PET reveals the beneficial effect of three‐phase enriched environment on post‐stroke memory deficits by enhancing plasticity of brain connectivity between hippocampus and peri‐hippocampal cortex

**DOI:** 10.1111/cns.14466

**Published:** 2023-09-27

**Authors:** Yun Lu, Mingcong Li, Yuming Zhuang, Ziyue Lin, Binbin Nie, Jianfeng Lei, Yuanyuan Zhao, Hui Zhao

**Affiliations:** ^1^ School of Traditional Chinese Medicine Capital Medical University Beijing China; ^2^ Beijing Key Lab of TCM Collateral Disease Theory Research Beijing China; ^3^ Beijing Engineering Research Center of Radiographic Techniques and Equipment, Institute of High Energy Physics Chinese Academy of Sciences Beijing China; ^4^ Core Facilities Center Capital Medical University Beijing China

**Keywords:** astrocyte‐neuron metabolic interaction, enriched environment, functional connectivity, learning and memory, MRI, PET

## Abstract

**Aim:**

The three‐phase enriched environment (EE) intervention paradigm has been shown to improve learning and memory function after cerebral ischemia, but the neuronal mechanisms are still unclear. This study aimed to investigate the hippocampal–cortical connectivity and the metabolic interactions between neurons and astrocytes to elucidate the underlying mechanisms of EE‐induced memory improvement after stroke.

**Methods:**

Rats were subjected to permanent middle cerebral artery occlusion (pMCAO) or sham surgery and housed in standard environment or EE for 30 days. Memory function was examined by Morris water maze (MWM) test. Magnetic resonance imaging (MRI) was conducted to detect the structural and functional changes. [^18^F]‐fluorodeoxyglucose (FDG) positron emission tomography (PET) was conducted to detect brain energy metabolism. PET‐based brain connectivity and network analysis was performed to study the changes of hippocampal–cortical connectivity. Astrocyte‐neuron metabolic coupling, including gap junction protein connexin 43 (Cx43), glucose transporters (GLUTs), and monocarboxylate transporters (MCTs), was detected by histological studies.

**Results:**

Our results showed EE promoted memory function improvement, protected structure integrity, and benefited energy metabolism after stroke. More importantly, EE intervention significantly increased functional connectivity between the hippocampus and peri‐hippocampal cortical regions, and specifically regulated the level of Cx43, GLUTs and MCTs in the hippocampus and cortex.

**Conclusions:**

Our results revealed the three‐phase enriched environment paradigm enhanced hippocampal–cortical connectivity plasticity and ameliorated post‐stroke memory deficits. These findings might provide some new clues for the development of EE and thus facilitate the clinical transformation of EE.

## Graphical abstract

The three‐phase enriched environment paradigm increased functional connectivity between the hippocampus and peri‐hippocampal cortex following cerebral ischemia, and reconfigured hippocampal–cortical network. In addition, EE had pleiotropic effects on modulating the expression of Cx43, GLUTs, and MCTs in the bilateral hippocampus and cortical regions, which are involved in astrocyte‐neuron metabolic coupling.

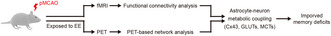

## 1 INTRODUCTION

Stroke is the main cause of adult disability in the world.^1^ Except for sensory and motor deficits, post‐stroke cognitive impairment increases the risk of dementia.^2^ In recent years, EE paradigms on the post‐stroke rehabilitation has attracted great attention.^3^, ^4^, ^5^ In general, EE intervention provides an environment that keeps animals in large cages containing tunnels, nesting material, a variety of toys to induce a complex combination of sensorimotor, cognitive, and social stimulation.^4^ Several studies previously demonstrated EE was beneficial to the memory formation via promoting synaptic plasticity of hippocampus in aged rat.^6^ We extended these findings and designed a three‐phase EE post‐stroke rehabilitation paradigm to provide various degrees of stimulation, which required rearranging EE cages periodically in line with acute, subacute, and convalescence phase after stroke. Our previous studies supported that this three‐phase EE paradigm was effective in improving post‐stroke cognitive function.^7^, ^8^ However, the underlying mechanisms have not been elucidated.

Accumulating evidences suggest that learning and memory function relies on neuronal network communication involving various brain regions.^9^ Specially, hippocampal and cortical regions have been implicated in a variety of learning memory‐related functions.^9^, ^10^, ^11^ Hippocampus is a cognitive region that is responsible for the spatial navigation and cognitive functions.^12^, ^13^ Entorhinal cortex innervates hippocampal areas and hippocampus sends direct projections back to the entorhinal cortex, which is involved in memory function.^12^ The main cortical projections from hippocampus are to anterior cingulate cortex and retrosplenial cortex, which participate in the cognitive processing of visuospatial information and memory processing in rats.^12^ Following stroke, lesions to hippocampal and cortical regions cause the limited efficiency of information transfer, leading to impaired memory.^14^ Notably, EE increased inter‐ and intra‐hemispheric functional connectivity in the retrosplenial cortex and cingulate cortex.^15^ However, few studies investigated the effect of EE on the hippocampal–cortical connectivity after cerebral ischemia.

Currently, functional MRI (fMRI) is by far the most popular method to investigate functional connectivity. However, accumulating evidence reported that one protocol cannot investigate the changes of information communication between brain regions, due to the complex interaction of biochemical and electrical signaling in brain activity.^16^ [^18^F]‐FDG PET imaging, which detects the cumulative consumption of glucose across the brain,^17^, ^18^ has been successfully applied to detect inter‐regional functional connectivity. Several studies demonstrated that brain functional topology is tightly coupled with cerebral metabolism,^19^, ^20^ and the changes in brain metabolism are associated with reorganized functional connectivity architecture.^21^ In particular, previous study constructed metabolic network and reported large‐scale abnormal brain connectivity in rats at the acute stage of ischemic stroke.^22^ However, these was lack of the study which provided insight into the metabolic network changes during convalescence phase of stroke. Thus, further investigations designed to explore the changes of connectivity within hippocampal–cortical metabolic networks during convalescence phase were essential for understanding EE‐induced learning and memory improvement.

Recently, astrocytes have been accepted to serve a key role in neuronal communication.^23^ Specifically, astrocytes deliver energy substrates to neuron via glucose transporters and monocarboxylate transporters, respectively. Moreover, astroglial gap junctions influence intercellular trafficking of glucose and its metabolites. All of these are essential for maintaining metabolic interactions between neurons and astrocytes, which serve a critical role in control learning and memory function.^24^ Although previous investigations described the beneficial effects of EE paradigm on neurons and astrocytes restorative ability following ischemic stroke,^25^ no conclusive data concerned the metabolic interactions between neurons and astrocytes following post‐ischemic EE intervention.

On these grounds, we aimed to investigate the EE‐induced plasticity of functional connectivity between hippocampus and peri‐hippocampal cortical regions through the combination of fMRI and PET imaging following stroke, with a particular emphasis on the astrocyte‐neuron metabolic interactions, including the gap junction protein Cx43, glucose transporters and monocarboxylate transporters in the hippocampus and cortical regions, all of which may gain new understanding of the mechanisms by which this EE paradigm promotes post‐stroke cognitive recovery.

## 2 MATERIALS AND METHODS

### 2.1 Animals

Eighty‐five adult male Sprague–Dawley rats weighing 300–320 g (aged 8 weeks) were purchased from Vital River Laboratory Animal Technology Company. Rats were housed in the SPF animal research center of Capital Medical University (SYXK [jing] 2018–0030) with controlled temperature under a 12‐h light/12‐h dark schedule. All experimental procedures were approved by Capital Medical University Animal Ethics Committee (Permit Number: AEEI‐2018‐052).

### 2.2 Cerebral ischemic model and experimental design

Focal cerebral ischemia was induced with permanent occlusion of the right middle cerebral artery (MCA) according to a previous method.^26^ Rats were anesthetized using isoflurane (5% for induction and 2% for maintenance) vaporized in a mixture of oxygen and air (1:1). The right common carotid artery (CCA), internal carotid artery (ICA), and external carotid artery (ECA) were separated. A monofilament nylon suture was inserted into the lumen of ICA, advanced for about 16–18 mm, and consequently blocked the middle carotid artery. During the surgery process, a rat monitoring system (Small Animal Instruments Inc.), involving cardiogram electrodes, was used to monitor the vital signs of rats during surgery. The body temperature was maintained at 37°C via a warm water circulation system.

Rats with successful pMCAO exhibited neurological symptoms such as circling and walking to the contralateral side, and were recruited in this study.^7^, ^8^ Rats displaying no distinct neurological symptoms and no tissue damage in T2WI maps were excluded from this study.

Rats with successful pMCAO were randomly divided into pMCAO + Standard Environment (MS) group and pMCAO + Enriched Environment (ME) group. Sham‐operated rats underwent the same surgery but no occlusion procedure, and were grouped into the Sham + Standard Environment (SS) group and Sham + Enriched Environment (SE) group.

There were two parts in this study. For part 1, rats were randomized selected by operators blinded to the treatment conditions for MRI scanning on 31st day after surgery and MWM test from 32nd to 36th day after surgery followed by histological study. For part 2, rats were used for MRI and [^18^F]‐FDG PET scanning on 31st day after surgery.

### 2.3 Three‐phase enriched environment paradigm

**FIGURE 1:**
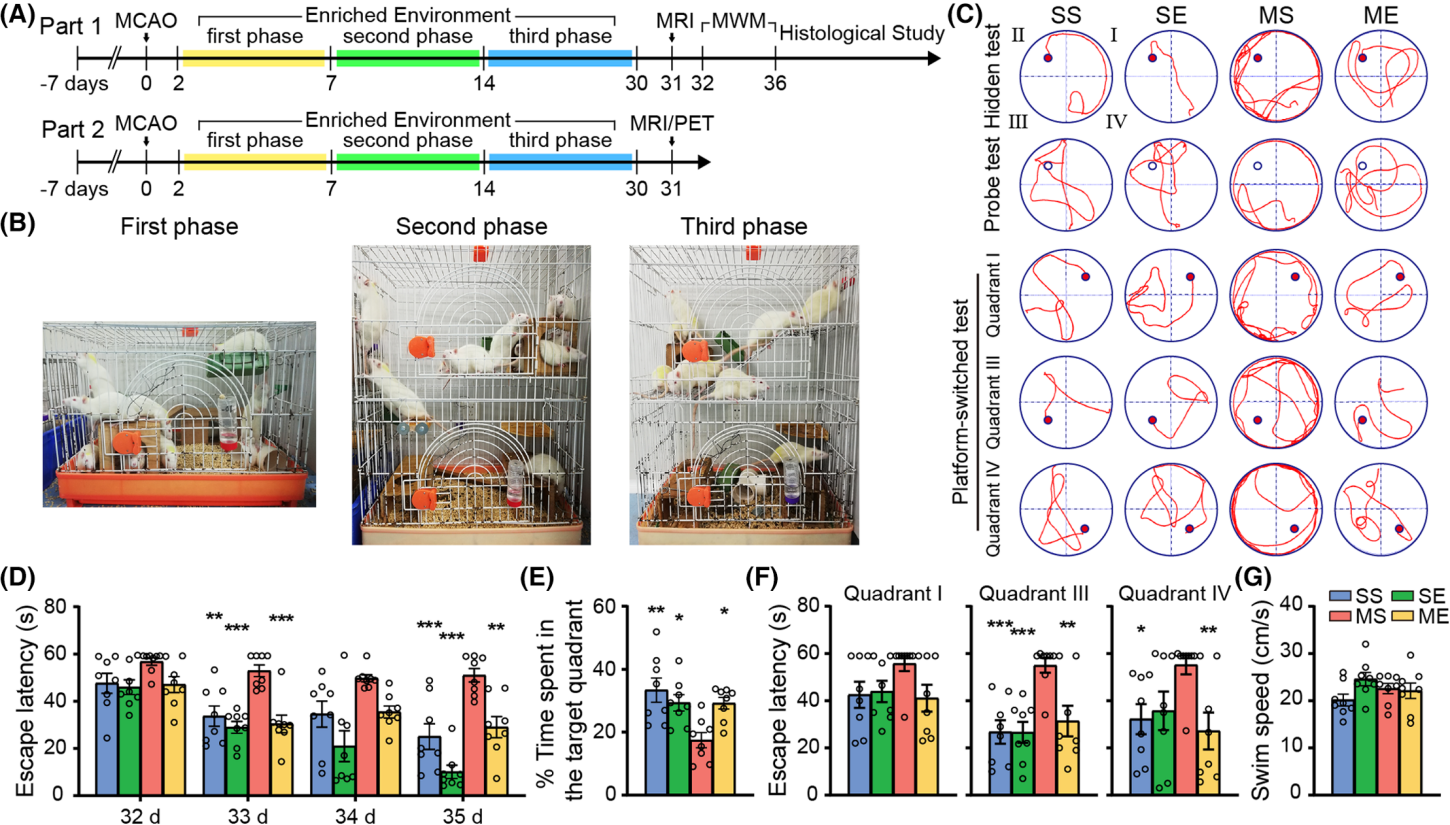
EE facilitated spatial memory improvement in pMCAO rats. (A) schematic timeline of the experiments. (B) Photograph of EE cages in three phases. (C) Typical traces of rats in the hidden platform test on 35th day, probe test and platform‐switched test on 36th day. (D) Escape latency of rats in the hidden platform test. (E) The percentage of time spent in target quadrant in probe test. (F) The time of rats to reach the platform switched in the quadrant I, III, and IV. (G) Swim speed of rats in hidden platform test on 35th day. Data were present as mean ± SEM, *N* = 8 rats per group. *p < 0.05, **p < 0.01, ***p < 0.001 versus the MS group.

Rats of SE and ME groups were caged in enriched environment (10–12 rats were housed together) for 12 h (8:00 p.m.–8:00 a.m.) and then caged in standard environment. The design of three‐phase EE intervention was described previously.^7^ In first phase (2–7 days after pMCAO), enriched environment cage (70 cm × 50 cm × 32 cm) contained toys and wheels to encourage sensorimotor stimulation and promote voluntary physical activity and social intercourse. In second phase (8–14 days after pMCAO), the enriched environment cage is arranged in two layers (70 cm × 50 cm × 64 cm), containing a variety of balance beams, swing boards, ladders, and tunnels to facilitate motor, sensory, and cognition activities. The position of these objects was altered every day to remain novel and complex. In third phase (15–30 days after pMCAO), the enriched environment cage is divided into three layers (70 cm × 50 cm × 64 cm), adding floating cabins, balance beams, ladders, and placing foods (melon seeds or peanuts) in different locations on the third layer, to induce extensive sensory, motor, social, and cognitive stimulation (Figure 1B).

Standard environment cage (40 cm × 30 cm × 20 cm) constituted only food box and water bottle. Rats were housed in sets of three per cage.

### 2.4 Magnetic resonance imaging protocols

Rats were anesthetized using isoflurane (5% for induction) vaporized in a mixture of oxygen and air (1:1) followed by the intramuscular injection dexmedetomidine (0.015 mg/kg). When rats were fully anesthetized, they were placed in the MRI scanning bed with the isoflurane concentration at 2% for maintenance. During the fMRI scanning process, the isoflurane concentration was decreased to 0.20%–0.25% to maintain a respiration rate of 60–85/min. As the respiratory rate increased to 90/min, the isoflurane concentration increased to 0.5%. A rat monitoring system (Small Animal Instruments Inc.), involving fiber optic oximetry sensor and cardiogram electrodes, was used to monitor the vital signs of rats. The body temperature was maintained at 37°C by a warm water circulation system.

MRI were performed on a 7.0 T PharmaScan Scanner (Bruker, Germany) on the 31st day after pMCAO. The coil used in the present study is rat surface coil (Bruker, Germany). T2‐weighted imaging (T2WI) was performed using a fast spin‐echo pulse sequence. Infarct volume was calculated via adding all infarct areas of individual slices (thickness 0.7 mm) using ImageJ.^7^ T2 relaxometry mapping was performed using a multi‐slice multi‐echo sequence. Diffusion tensor imaging (DTI) was performed using an axial single‐shot spin echo‐planar imaging sequence.^8^ For fMRI, Blood‐oxygen‐level‐dependent (BOLD) imaging was performed using an EPI‐SE‐FOVsat sequence. The details of each sequence were listed in Table S6. Paravision version 5.1 software (Bruker, Germany) was used to reconstructed DTI‐derived apparent diffusion coefficient (ADC) maps. T2 and ADC value in the bilateral hippocampus (Hip), entorhinal cortex (Ent), cingulate cortex (Cg), and retrosplenial cortex (RSC) was obtained.^27^

### 2.5 fMRI image preprocessing

Processing of the fMRI data was constructed with spmratIHEP software based on statistical Parametric Mapping software (SPM8) and Resting State fMRI Data Analysis Toolkit V1.8 software (REST). A self‐adaption template image was firstly constructed iteratively from these MCAO images. Then, the intercranial tissue of this template image was extracted manually and saved as a binary mask image. Spatially standardizing the mask image into Paxinos space and carrying the self‐adaption template image along. Finally, all the images were normalized to the standardized self‐adaption template. Sequential data processing steps included: slice‐timing adjustment, realignment and correction for head‐motion, spatial normalization to the standard rat brain atlas in Paxinos space,^28^ smoothing with an isotropic Gaussian kernel (FWHM = 1 mm), detrending, and filtering (0.01–0.1 Hz). Data were excluded if head movements exceeded 1.0 mm of maximum translation in the *x*, *y*, or *z* directions or 2.0 of maximum rotation about the three axes.

### 2.6 Functional connectivity analysis

The functional connectivity analysis was performed using REST software. Seed regions were placed at the right and left hippocampus, respectively. These regions were selected based on the decreased ADC in these areas after EE intervention. Pearson's correlation was then carried out between each voxel in each seed and the time series of every voxel in the brain to obtain FC maps for each rat. One‐way ANOVA was performed to identify significant differences in FC values among SS, SE, MS, and ME groups followed by post hoc analysis using two‐sample *t* test. All results were corrected by Gaussian random field (GRF) theory (voxel‐level, *p* < 0.005; cluster‐level, cluster size >50).

### 2.7 PET/CT imaging procedures

PET measurements were conducted on the 31st day after pMCAO. Rats accepted an intravenous injection with 18.5 MBq (500 μCi) of [^18^F] FDG into the tail vein, and experienced an uptake period for 1 h. Rats were anesthetized using isoflurane (5% for induction; 2% for maintain) vaporized in a mixture of oxygen and air (1:1). T2WI scanning was performed previously before PET scanning. PET/CT scanning was performed by a high‐resolution micro‐PET/CT scanner (Siemens). Inveon Research Workplace software (Siemens) was used to register and fuse PET and T2WI images. Standard uptake value (SUV) in bilateral hippocampus, entorhinal cortex, cingulate cortex, retrosplenial cortex, and cerebellum was obtained. Cerebellum served as a reference region. PET result was present as Standard uptake value ratio (SUVR) generated by normalizing SUV with uptake in cerebellum.^29^

### 2.8 Construction of hippocampal–cortical metabolic network

Statistical analysis of [18F] FDG images was performed by SPM8 (Wellcome Department of Clinical Neurology, UK) with the spmratIHEP plugin.^28^, ^30^, ^31^ Individual PET images were spatially normalized into the rat [^18^F]‐FDG PET template based on Paxinos & Watson atlas and smoothed using a Gaussian kernel of a 2 × 2 × 4 mm^3^ full‐width‐at‐half‐maximum (FWHM).

For metabolic network analysis, 10 volumes of interest (VOIs), including hippocampus, dentate gyrus, entorhinal cortex, cingulate cortex, and retrosplenial cortex in the bilateral hemispheres, were selected to generate hippocampal–cortical network based on a rat brain template.^28^ Regional [^18^F]‐FDG uptake in VOIs was calculated. Pearson's correlation coefficients between pair‐wise ROIs were obtained through inter‐subject analysis. Matrices (left and right hemispheres, 10 × 10 matrices) were constructed, where correlation coefficients used to represent the strength of each brain connectivity.^32^

### 2.9 Hippocampal–cortical connectivity analysis

Permutation test for 10,000 times was performed to analyze the difference of brain connectivity between two groups.^22^ In every time, we randomly assigned the SUV of each VOIs into two new groups, whose sizes were same as the original groups. Then, we calculated the brain connectivity of pair‐wise regions and constructed a new group‐level metabolic network. After 10,000 times, we obtained 10,000 random inter‐group differences in brain connectivity. The *p* value was obtained according to the percentile position of the real inter‐group difference in the corresponding permutation distribution. The false discovery rate (FDR) was applied to correct for multiple comparisons at a threshold of *p* < 0.05.^22^

### 2.10 Graph theory‐based connectivity analysis

Graph theoretical analysis was used to identify brain connectivity patterns through the Brain Connectivity Toolbox (https://sites.google.com/site/bctnet/).^22^ Global network property was evaluated by global efficiency (*E*_global_), which was the inverse of all the network nodes' average shortest path length.^22^ Permutation test repeated for 10,000 times was used to analyze the difference of network properties between two groups.^22^ *p* < 0.05 was considered statistically significant.

### 2.11 Morris water maze test

Spatial learning and memory function was assessed with Morris water maze from 32nd to 36th day after surgery. MWM consisted of hidden platform test, probe test, and switched platform test. Hidden platform test required rats to learn the spatial location of a hidden platform from 32nd to 35th day after surgery. Probe test required rats to find the location when platform was removed on 36th day after surgery. Switched platform test required rats to find the platform switched to other quadrants on 36th day after surgery. Morris test assay was conducted as previously reported.^8^ The platform was placed in the center of quadrant II in hidden platform test. A video camera was used to record swimming traces. An image analyzer software (Jiliang, China) was used to calculate escape latency in hidden platform test, the percentage of time spent in target quadrant in probe test, and escape latency of rats in switched platform test, as described previously.^8^

### 2.12 Tissue processing

There were two part of experiment. Thirty‐two rats underwent sequential MRI imaging and MWM test were weighted and deeply anesthetized. Random four rats per group were transcardially perfused and the brains were isolated and post‐fixed as previously described.^7^ Brain blocks (Bregma −2.2 to −4.5 mm) were cut, processed, and embedded in paraffin. A series of 5 μm‐thick coronal sections were sliced from the blocks for Immunohistochemistry. The rest of four rats per group were dissected. Bilateral hippocampus and entorhinal cortex tissues were separated and frozen in liquid nitrogen for Immunoblotting analysis.^33^, ^34^

In the second part of the study, 32 rats recruited for the PET scanning were weighted and deeply anesthetized following PET scanning. The brains were quickly dissected and stored for related research.

### 2.13 Immunohistochemistry staining and image analysis

Double‐label immunofluorescence staining and image analysis were performed as previously described.^7^ Sections were immersed in 5% goat serum for 1 h and then incubated overnight at 4°C with primary antibodies, including anti‐connexin 43 (Cx43, Abcam, ab66151, 1:200), anti‐glial fibrillary acidic protein (GFAP, Millipore, MAB360, 1:600). Secondary antibodies conjugated with Alexa Fluor 488 (SouthernBiotech, 1036–02, 1:400) and 594 (SouthernBiotech, 4030–03, 1:400) were diluted in blocking buffer and incubated for 2 h at 37°C. The brain slices were attached to coverslips and mounted with VECTASHIELD Mounting Medium (Vector Laboratories, USA) containing correct dilution of DAPI (SouthernBiotech, USA). All slices were viewed using a fluorescent microscopy (Nikon, Japan). Non‐overlapping regions (3 for right peri‐hippocampal cortex and bilateral Cornus Amonis area 1 (CA1); 2 for bilateral dentate gyrus (DG)) were randomly collected. All images were analyzed with NIS‐Elements BR Analysis software (Nikon, Japan). The positive expression of GFAP/Cx43 was represented with integrated optical density (IOD).^35^ Data acquisitions and analyses were performed in blind conditions of the experiments.

### 2.14 Immunoblotting analysis

Experiments were performed as previously reported.^7^ Protein concentrations were established using a BCA protein assay kit (Applygen, China, cat. No. P1511). The following antibodies were used in the immunoblotting experiments: synaptophysin (SYN, EPITOMICS, 1870–1, 1:20000), growth associated protein‐43 (GAP‐43, EPITOMICS, 2259–1, 1:2000), glucose transporter 1 (GLUT1, Millipore, MABS132, 1:6000), glucose transporter 3 (GLUT3, Abcam, ab15311, 1:8000), monocarboxylate transporter 2 (MCT2, Abcam, ab224627, 1:10000), monocarboxylate transporter 4 (MCT4, Santa, Sc‐376,140, 1:8000), and GAPDH (1:40000; Genetex, GTX100118). Protein bands were visualized by ChemiDoc XRS+ Imaging System (Bio‐Rad, USA). Quantitation of protein intensities was performed using ImageJ software. Relative intensity of each protein was normalized to GAPDH. Data acquisitions and analyses were performed in blind conditions of the experiments.

### 2.15 Statistical analysis

SPSS 26.0 and Matlab 2014a were used for all analysis. Data distribution was tested for normality using the Shapiro–Wilk test. Datasets with normal distribution and equal within‐group variances (from hidden‐platform and probe test, ADC value, relative SUV, GFAP/Cx43 expression and Immunoblotting results) were tested by two‐way ANOVA followed by post hoc analysis using Bonferroni. Datasets with normal distribution but unequal population variances (from MRI results) were tested by Tamhane test. Data that did not follow the normal distribution (from the switched platform test and T2 value) were analyzed via a non‐parametric Kruskal–Wallis test. Differences were considered statistically significant at *p* < 0.05.

For fMRI data, the analysis procedure was mentioned in section Functional Connectivity Analysis.

## 3 RESULTS

### 3.1 EE improved spatial learning and memory in pMCAO rats

Total 32 rats (eight rats per group) were recruited in the part 1 of the study. In the hidden platform test, two‐way repeated measures ANOVA yielded significant main effects of time and EE, and time × EE interaction on the escape latency. Post hoc analysis indicated escape latency increased in MS group in comparison with SS group on 33rd and 35th day (Figure 1D). ME group displayed a significant decrease of escape latency compared with MS group on 33rd and 35th day (Figure 1D).

In the probe test, two‐way ANOVA showed prominent MCAO × EE interaction on the time spent in target quadrant. Post hoc analysis revealed that MS group spent less time in target quadrant in comparison with SS group (Figure 1E). However, ME group showed increased percent of time spent in the target quadrant in comparison to MS rats (Figure 1E).

In the platform‐switched test, our results revealed increased time for MS group to find the switched platform in quadrant III and IV compared with SS group (Figure 1F). ME group rats spent less time to locate the platform in quadrant III and IV in comparison with MS group (Figure 1F).

There was no significant difference in swimming speed among four groups, which suggested that no motor deficits may be affecting water maze detection (Figure 1G).

### 3.2 EE ameliorated microstructural injuries of the bilateral hippocampus and peri‐hippocampal cortex in pMCAO rats

**FIGURE 2:**
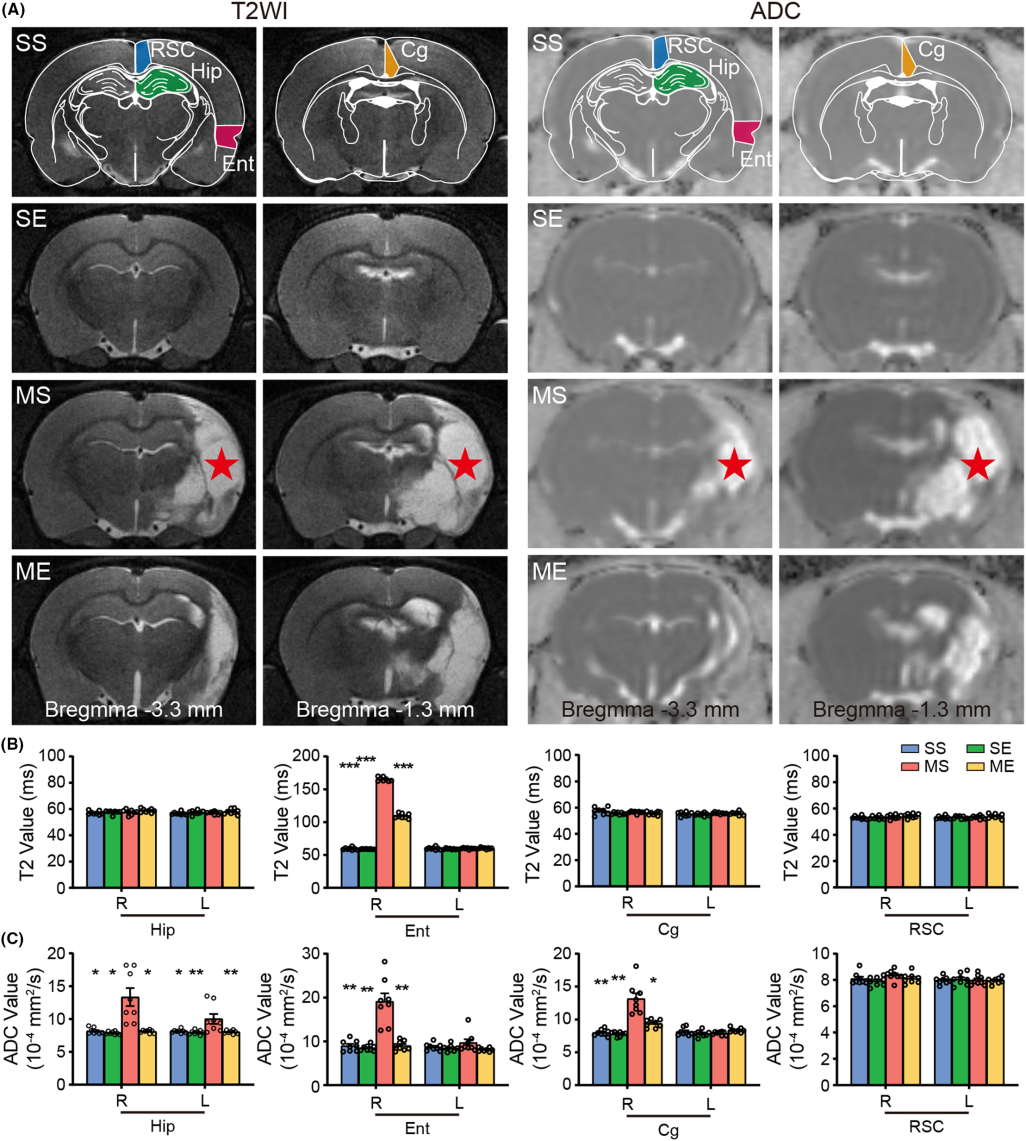
EE protected microstructural integrity of hippocampus and peri‐hippocampal cortex in pMCAO rats. (A) Representative T2WI, ADC images. Schematic illustration of hippocampus (green), entorhinal cortex (red), cingulate cortex (yellow), and retrosplenial cortex (blue) of rat brain diagram. Red star represented infarct areas. Quantitative analysis of T2 (B) and ADC (C) in the bilateral hippocampus, entorhinal cortex, cingulate cortex and retrosplenial cortex. *N* = 8 rats per group. *p < 0.05, **p < 0.01, ***p < 0.001 vs. the MS group. Cg, cingulate cortex; Ent, entorhinal cortex; Hip, hippocampus; L, left; R, right; RSC, retrosplenial cortex.

Coronal T2WI images displayed abnormal hyperintense signal in the right MCA territory (Figure 2A).There was no significant difference of infarct volume between MS and ME groups (Figure S1). Notably, T2 value in the right entorhinal cortex of MS group significantly increased compared to that of SS group (Figure 2B). After exposed to EE, ME group showed decreased T2 value in the right entorhinal cortex compared to MS group (Figure 2B).

DTI‐derived ADC results revealed abnormally increased ADC of the right hippocampus, entorhinal cortex, cingulate cortex, and the left hippocampus in MS group compared with that of SS group (Figure 2C). ME group showed decreased ADC in the corresponding regions in comparison with MS group (Figure 2C).

### 3.3 EE enhanced the hippocampal functional connectivity with peri‐hippocampal cortex in pMCAO rats

**FIGURE 3:**
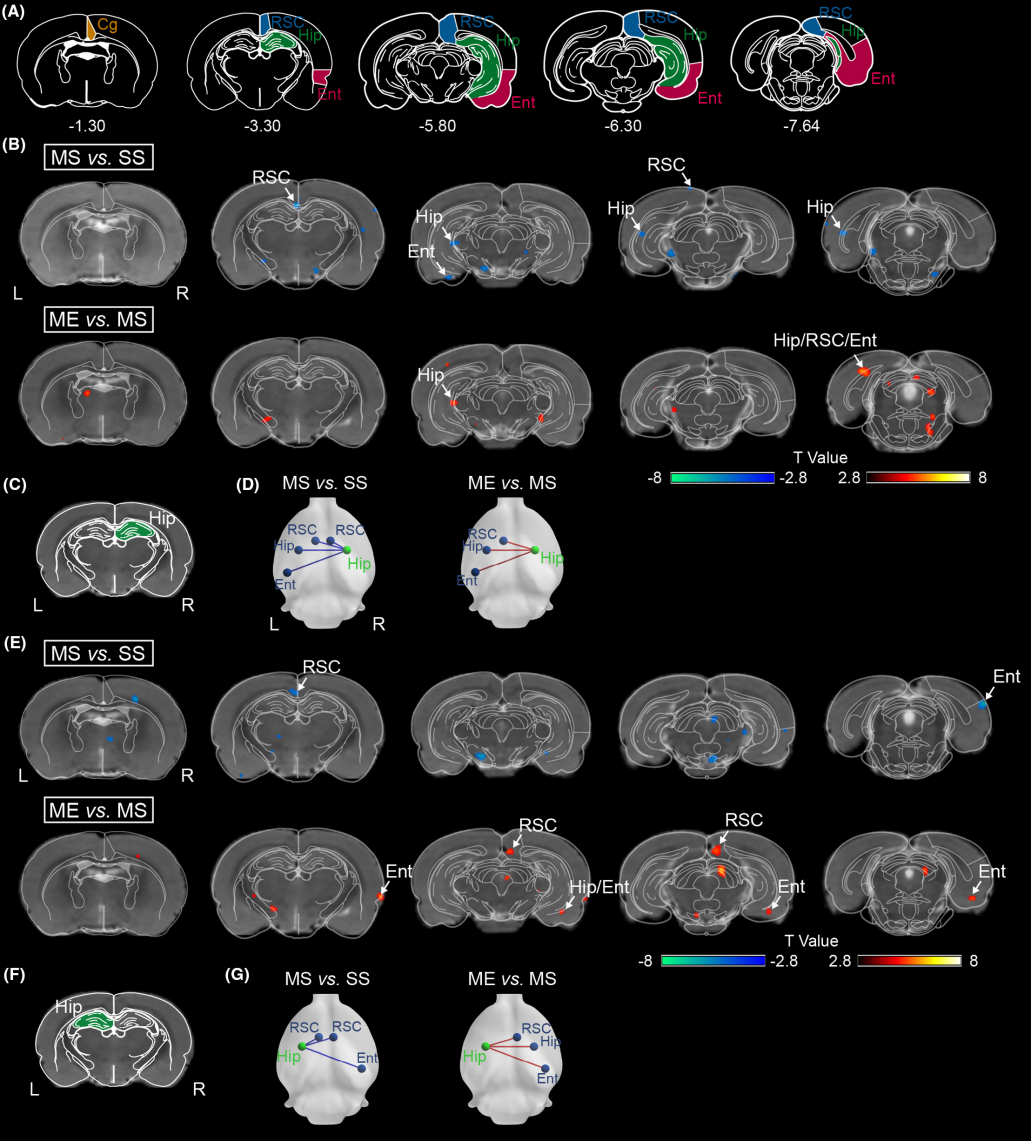
EE activated bilateral hippocampal functional activity with peri‐hippocampal cortex in pMCAO rats. (A) Schematic illustration of hippocampus (green), entorhinal cortex (red), cingulate cortex (yellow), and retrosplenial cortex (blue) of rat brain diagram. Distribution of significantly different FC with (B) the right and (E) the left hippocampus as the seed was overlaid in 3D brain surfaces. Seed regions for FC analysis are (C) the right and (F) the left hippocampus, overlaid on a histological template. Contrast maps locating the distribution of significant differences in FC with (D) the right and (G) the left hippocampus. Red lines indicate increased connectivity and blue lines indicate decreased connectivity. Cg, cingulate cortex; Ent, entorhinal cortex; Hip, hippocampus; L, left; R, right; RSC; retrosplenial cortex.

When the seed was located in the right hippocampus, the results are illustrated in Figure 5 and the data for different clusters are shown in Table S1. Compared to SS group, MS group showed significant decreased right hippocampal FC in several regions, involving the left hippocampus, entorhinal cortex, and the bilateral retrosplenial cortex (Figure 3B). After exposed to the EE, the FC in the right hippocampus was increased in the above regions, such as the left hippocampus, entorhinal cortex and retrosplenial cortex (Figure 3B).

When the seed was located in the left hippocampus, the results are exhibited in Figure 6 and the data for different clusters are shown in Table S2. Our results revealed that the left hippocampal FC of MS group was decreased in several brain regions, including the right entorhinal cortex and the bilateral retrosplenial cortex, in comparison with SS group (Figure 3C). After EE intervention, the FC between the left hippocampus and the above regions, such as the right hippocampus, entorhinal cortex, and retrosplenial cortex, was significantly increased (Figure 3C).

### 3.4 EE enhanced glucose metabolism of the bilateral hippocampus and peri‐hippocampal cortex in pMCAO rats

**FIGURE 4:**
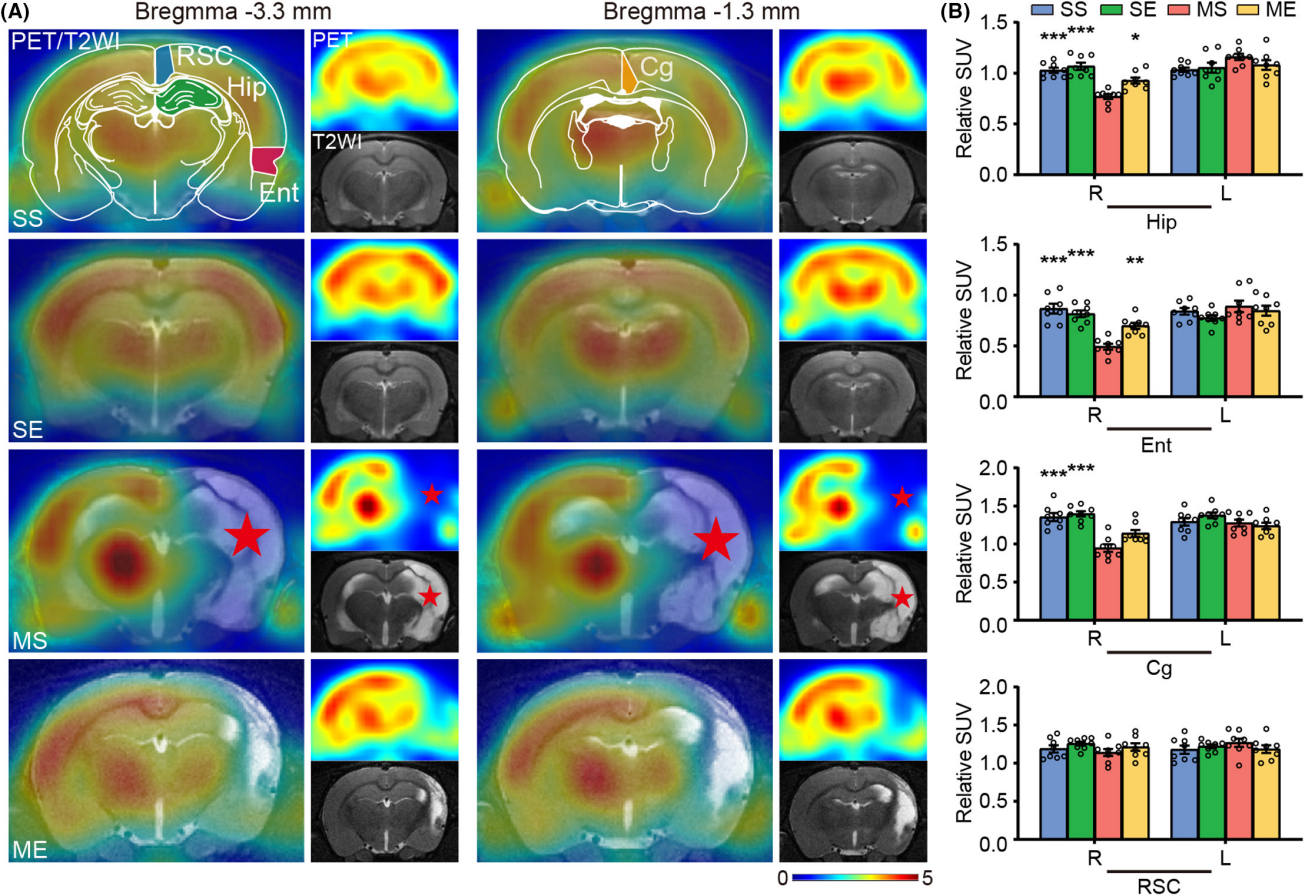
EE enhanced glucose metabolism of the right hippocampus and peri‐hippocampal cortex. (A) Representative PET/T2WI, PET, and T2WI images. Schematic illustration of hippocampus (green), entorhinal cortex (red), cingulate cortex (yellow) and retrosplenial cortex (blue) of rat brain diagram. Red star represented infarct areas. (B) Quantitative analysis of SUVR in the bilateral hippocampus, entorhinal cortex, cingulate cortex and retrosplenial cortex. Data were present as mean ± SEM, *N* = 8 rats per group. *p < 0.05, **p < 0.01, ***p < 0.001 vs. the MS group. Cg, cingulate cortex; Ent, entorhinal cortex; Hip, hippocampus; L, left; R, right; RSC, retrosplenial cortex.

Total 32 rats (eight rats per group) were recruited in the part 2 of the study. The cerebral glucose metabolism was measured with [^18^F]‐FDG PET imaging. Two‐way ANOVA yielded significant main effect of EE and EE × pMCAO interaction on the regional SUVR. Post hoc analysis revealed that MS group showed decreased SUVR in the right hippocampus, entorhinal cortex, and cingulate cortex compared to the SS group (Figure 4B). EE increased SUVR in the right hippocampus and entorhinal cortex in the cerebral ischemic rats (Figure 4B).

### 3.5 PET‐based connectivity analysis revealed that EE strengthened hippocampal–cortical connectivity in pMCAO rats

**FIGURE 5:**
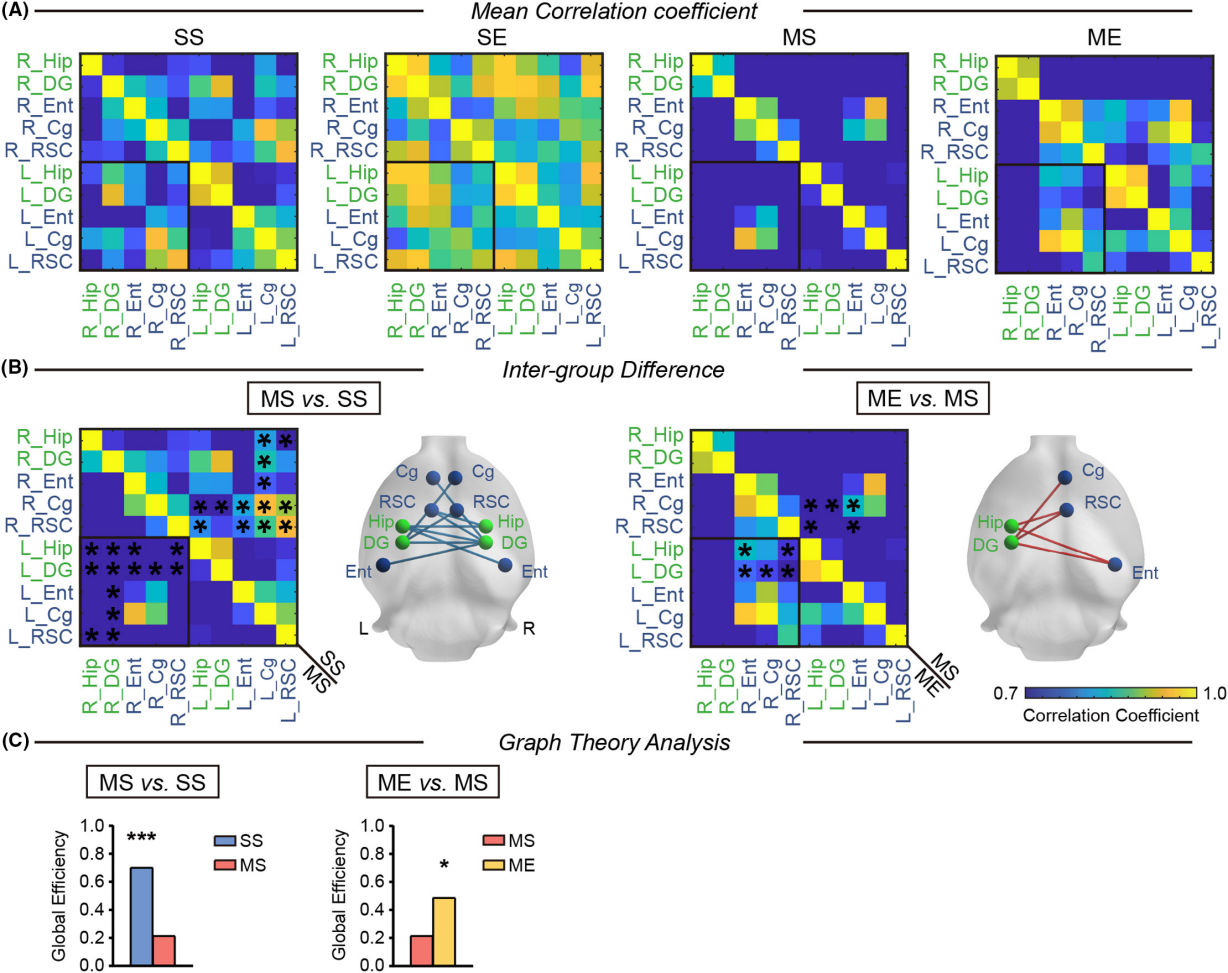
PET‐based connectivity analysis revealed EE‐induced strengthened hippocampal–cortical connectivity in pMCAO rats. (A) PET‐derived cross‐correlation matrices with the yellow‐blue color gradient showing region‐to‐region connectivity (corrected with FDR). (B) Significant difference of the connectivity between two regions for MS versus SS and ME versus MS. Contrast maps locating the distribution of significant differences in connectivity. (C) Summary graph depicting global efficiency for MS versus SS and ME versus MS. *N* = 8 rats per group. *p < 0.05, ***p < 0.001 versus the MS group. Cg, cingulate cortex; Ent, entorhinal cortex; Hip, hippocampus; L, left; R, right; RSC, retrosplenial cortex.

Our results revealed that MS rats showed a large‐scale decreased brain connectivity within hippocampal–cortical network compared with SS group (Figure 5B, Table S3). ME rats exhibited increased brain connectivity between the left hippocampus and the right cortex, including entorhinal cortex and retrosplenial cortex, compared with MS group (F igure 5B, Table S3).

### 3.6 Impact of EE on the topological properties of the hippocampal–cortical network in pMCAO rats

Inter‐group differences of the global network parameters are listed in Tables S4 and S5. Global graph theory analysis revealed MS group rats displayed decreased *E*_global_ of hippocampal–cortical network in comparison with SS group (Figure 5C). EE intervention increased *E*_global_ in rats following ischemic stroke (Figure 5C).

### 3.7 EE increased synaptic plasticity and axon growth of the bilateral hippocampus and peri‐hippocampal cortex in pMCAO rats

**FIGURE 6:**
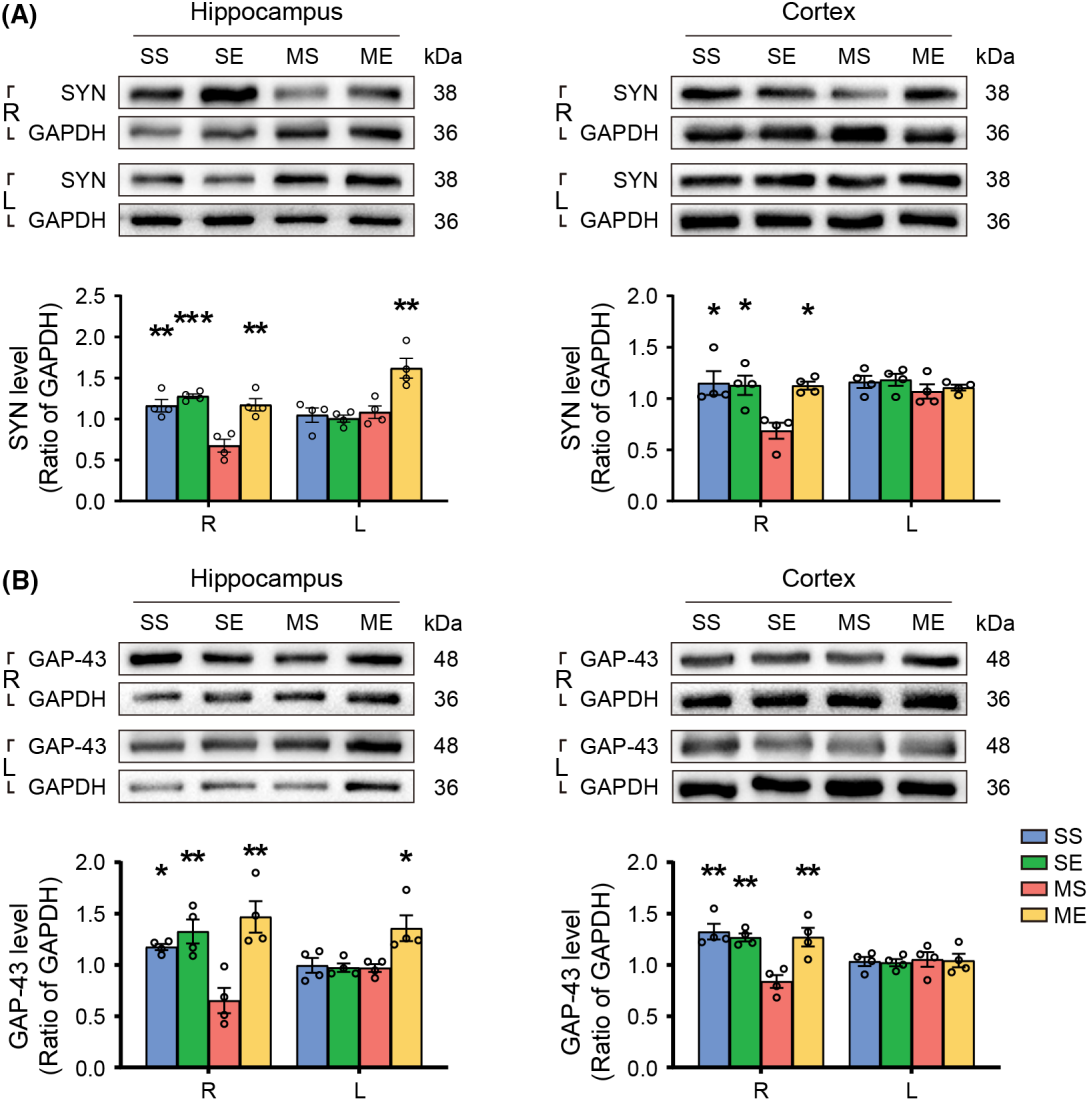
EE increased synaptic plasticity and axon growth in pMCAO rats. Representative images and Western blot analysis for (A) SYN and (B) GAP‐43. The protein level was quantified using GAPDH as the loading controls. Data were present as mean ± SEM, *N* = 4 rats per group. *p < 0.05, **p < 0.01, ***p < 0.001 versus the MS group. R, right; L, left.

We assessed synaptogenesis and axonal growth by the expression of SYN and GAP‐43, respectively. Two‐way ANOVA yielded main effect of EE intervention and EE × pMCAO interaction on the level of SYN and GAP‐43 in the bilateral hippocampus and peri‐hippocampal cortex. Post hoc analysis showed decreased level of SYN and GAP‐43 in the right hippocampus and peri‐hippocampal cortex (Figure 6A,B). EE intervention increased level of SYN and GAP‐43 in the bilateral hippocampus and the right peri‐hippocampal cortex after stroke (Figure 6A,B).

### 3.8 EE regulated the expression of GFAP and Cx43 in the hippocampus and peri‐hippocampal cortex in pMCAO rats

**FIGURE 7:**
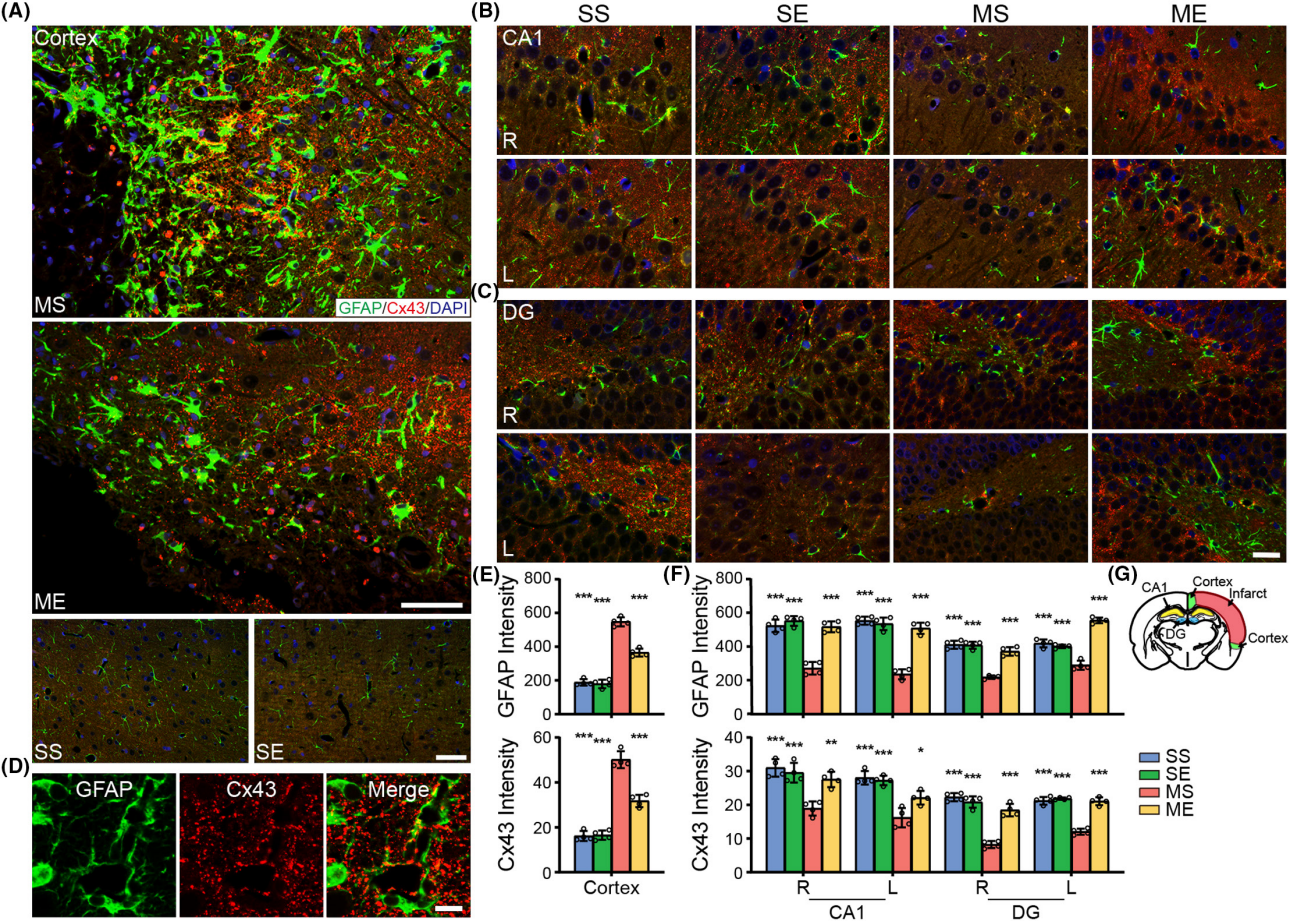
EE regulated the protein expressions of GFAP and Cx43 in pMCAO rats. Representative double immunofluorescence images of sections from (A) the peri‐hippocampal cortex, and the bilateral hippocampal (B) CA1 and (C) DG. Scale bars = 50 μm (A), and 20 μm (B, C). (D) Double labeling of GFAP (green) and Cx43 (red) in the peri‐hippocampal cortex. Scale bar = 10 μm. Quantitative data in integrated optical density (IOD) of GFAP and Cx43 in (E) the right peri‐hippocampal cortex, and (F) the bilateral CA1 and DG. (G) Schematic illustration of infarct (red), peri‐hippocampal cortex (green), CA1 (yellow) and DG (blue) of rat brain diagram. Data were present as mean ± SEM, *N* = 4 rats per group. *p < 0.05, **p < 0.01, ***p < 0.001 versus the MS group. CA1, Cornus Amonis area 1; DG, dentate gyrus; R, right; L, left.

Cx43, one of astrocytic gap‐junction channels, allows glucose and its energetic metabolites for intercellular delivery.^36^ Two‐way ANOVA yielded significant main effect of EE intervention and EE × pMCAO interaction on expression level of GFAP and Cx43 in the right peri‐hippocampal cortex as well as the bilateral hippocampal CA1 and DG regions. Post hoc analysis showed GFAP and Cx43 expression of MS group increased in the peri‐hippocampal cortex (Figure 7E), and decreased in the bilateral CA1, DG (Figure 7F) compared with that of SS group. EE intervention decreased expression of GFAP and Cx43 in the peri‐hippocampal cortex (Figure 7E), and increased expression in the bilateral CA1, DG (Figure 7F).

### 3.9 EE regulated the metabolic interactions between astrocytes and neurons of hippocampus and peri‐hippocampal cortex in pMCAO rats

**FIGURE 8:**
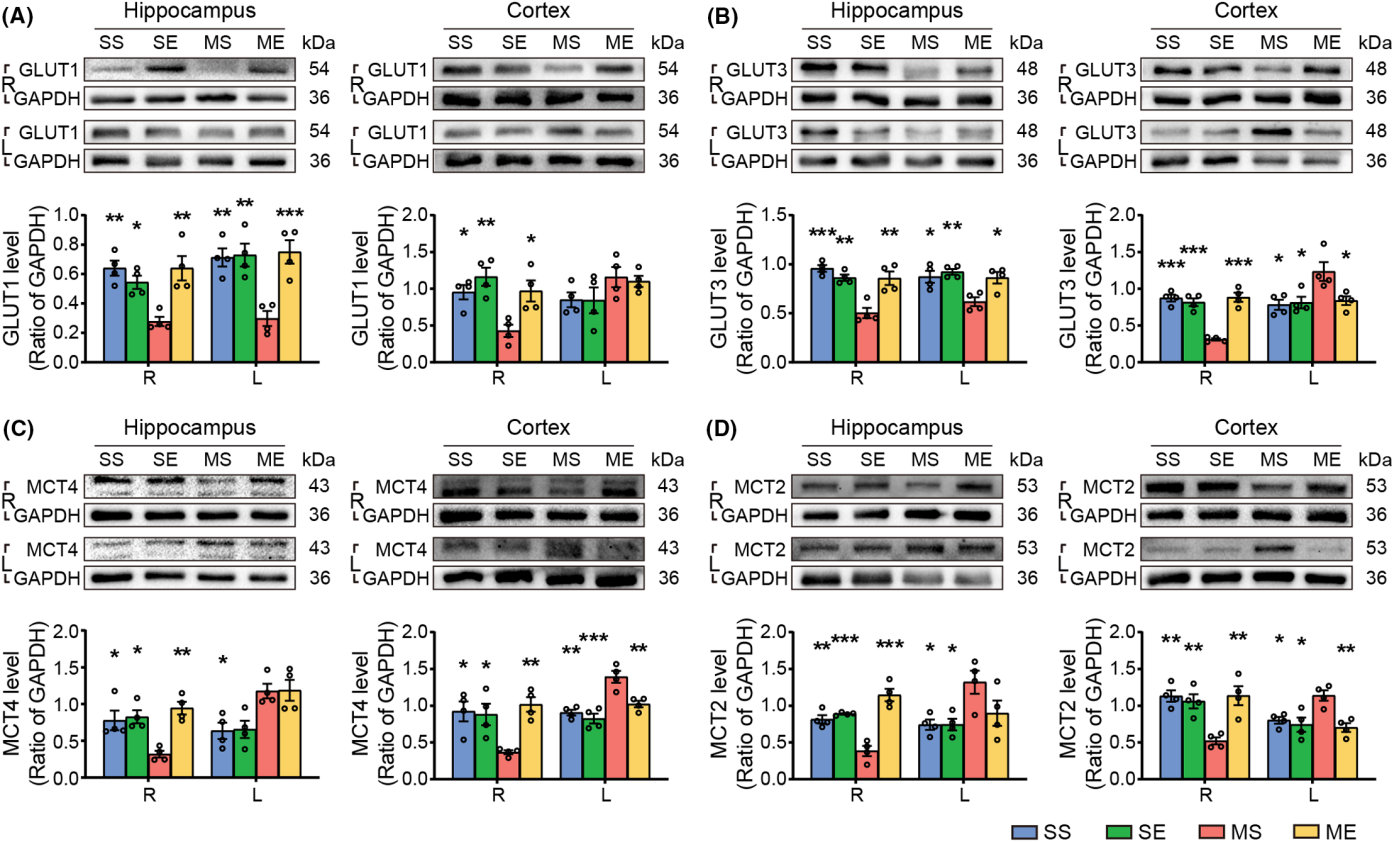
EE regulated glucose transporters and monocarboxylate transporters of the hippocampus and peri‐hippocampal cortex in pMCAO rats. Representative images and Western blot analysis for (A) GLUT1, (B) GLUT3, (C) MCT4, and (D) MCT2. The protein level was quantified using GAPDH as the loading controls. Data were present as mean ± SEM, *n* = 4 rats per group. *p < 0.05, **p < 0.01, ***p < 0.001 versus the MS group. R, right; L, left.

The glucose and lactate transporters serve a critical role in the energy metabolic cooperativity between astrocyte and neuron.^37^ Two‐way ANOVA yielded significant main effect of EE and EE × pMCAO interaction on the expression of GLUT1/GLUT3. Post hoc analysis revealed MS group showed decreased GLUT1/GLUT3 level in the bilateral hippocampus and the right peri‐hippocampal cortex (Figure 8A,B). EE intervention increased the GLUT1/GLUT3 expression in the bilateral hippocampus and the right peri‐hippocampal cortex following cerebral ischemia (Figure 8A,B).

Additionally, two‐way ANOVA revealed significant main effect of EE intervention and EE × pMCAO interaction on the expression of MCT4 and MCT2. Post hoc analysis revealed MS group exhibited decreased level of MCT4/MCT2 in the right hippocampus and peri‐hippocampal cortex, and increased MCT4/MCT2 expression in the left hippocampus and peri‐hippocampal cortex (Figure 8C,D). EE intervention increased expression of MCT2 and MCT4 in the right hippocampus and peri‐hippocampal cortex, and decreased the MCT4/MCT2 expression in the left peri‐hippocampal cortex following cerebral ischemia (Figure 8C,D).

## 4 DISCUSSION

In this study, we found that the three‐phase enriched environment paradigm ameliorated hippocampus and peri‐hippocampal cortical microstructural lesion, benefited glucose metabolism, and improved spatial learning and memory in pMCAO rats. Furthermore, the present study demonstrated that EE increased functional connectivity between the hippocampus and peri‐hippocampal cortex following cerebral ischemia, and reconfigured hippocampal–cortical network. Indeed, EE had pleiotropic effects on modulating the expression of gap junction protein Cx43, glucose transporters and monocarboxylate transporters in the bilateral hippocampus and peri‐hippocampal cortex. Such proteins are involved in astrocyte‐neuron metabolic cooperation, suggesting EE might enhance the metabolic interactions between astrocyte and neuron, which was essential for the plasticity of hippocampal–cortical connectivity after ischemic stroke.

MWM test revealed that EE decreased escape latency in the hidden‐platform test, elevated time spent in the target quadrant in the probe test, and decreased escape latency in the switched‐platform test following stroke. Such results are consistent with previous investigations.^7^ Based on these results, our findings illustrated that the reorganization of the hippocampus and peri‐hippocampal cortex is essential for improving cognitive function following post‐stroke EE intervention.

MRI is sensitive to tissue damage induced by stroke.^38^ Here, T2WI showed a wide range of infarct in right MCA‐supply areas. Our observation revealed that unilateral stroke induced significant tissue lesion, evidenced by the increased T2 value in MS group. EE decreased T2 value in ischemic rats, which indicated positive effect of EE on the post‐stroke structural injury. In addition, DTI‐derived ADC values are sensitive to changes of microstructural integrity.^39^, ^40^ In general, the elevated ADC values are possibly due to neuronal degeneration, while decreased ADC values represent microstructural remodeling after stroke.^41^ Our results displayed augmented ADC signal intensity in the bilateral hippocampus and peri‐hippocampal cortex (entorhinal cortex, cingulate cortex) in pMCAO rats. However, EE significantly decreased ADC value in corresponding regions after stroke. MRI data herein illustrated that unilateral MCA occlusion degraded the microstructure of the hippocampus and peri‐hippocampal cortex. However, EE provided benefits for the microstructural restoration in the hippocampus and peri‐hippocampal cortex following ischemia.

The hippocampus is known to coordinate with cortical regions in order to facilitate memory encoding and storage.^42^ Previous studies have reported the lack of neuronal communication between hippocampus and cortical areas disrupted memory function.^14^ Moreover, we utilized a fMRI‐based FC analysis to explore the effect of EE on the functional connectivity between hippocampus and cortex following cerebral ischemia. Herein, our results revealed that cerebral ischemia caused decreased inter‐hemispheric functional connectivity between hippocampus and peri‐hippocampal cortex (entorhinal cortex and retrosplenial cortex). Of note, EE was beneficial to the reformation of hippocampal–cortical functional connectivity following ischemic stroke. In healthy rats, EE was observed to strengthen functional connectivity between hippocampus and cingulate/retrosplenial cortex,^15^ which might be associated with the repair of the memory dysfunction.

[^18^F]‐FDG PET imaging provide relatively stable metabolic information correlating with neuronal activity.^16^ Based on the correlation, [^18^F]‐FDG PET has been applied to detect neural activity and study brain connectivity. Here, our results showed hypometabolism in the right hippocampus, entorhinal cortex, and cingulate cortex. Notably, EE elevated glucose metabolism in the right hippocampus and entorhinal cortex. A clinic study has reported a positive correlation between regional glucose metabolism and cognitive function.^43^ EE‐induced elevated level of neuronal activity might be the mechanism for memory improvement after stroke.

The elevated level of neuronal activity leads to the selection of that neuron into a circuit of memory recovery.^44^ For this reason, we utilized a PET‐based inter‐group network analysis to explore the effect of EE on hippocampal–cortical connectivity. The data revealed that unilateral MCA occlusion caused wide‐ranging decreased connectivity between the bilateral hippocampus, and between the hippocampus and cortical regions. Of note, EE increased inter‐hemispheric connectivity between the left hippocampus and right peri‐hippocampal cortex (entorhinal cortex and retrosplenial cortex), which was in line with the observation in FC analysis. Combination of fMRI and PET imaging revealed that EE strengthened the inter‐hemispheric functional connectivity between left hippocampus and right cortex. We speculated that EE‐induced strengthened inter‐hemispheric brain connectivity between hippocampus and peri‐hippocampal cortex might be part of a compensatory strategy that left hippocampus participated in memory functional repair.

Furthermore, we analyzed network parameters based on graph theory. Our results agree with other studies showing focal cerebral ischemia disturbed network as evidenced by decreased global efficiency.^22^ While EE increased global efficiency of hippocampal–cortical network. It is generally accepted that the enhanced global efficiency is closely related to improved network's ability for parallel information transfer between nodes.^45^ Overall, network features presented here might indicate a reorganized hippocampal–cortical network after EE intervention. Although not yet completely understood, results herein illustrated that EE benefited hippocampal–cortical network reorganization that might be essential to rehabilitate cognition function following ischemic stroke.

Specifically, we detected the expression of growth‐associated proteins, and found EE promoted the expression of the GAP‐43 and synaptogenesis marker SYN in the bilateral hippocampus and right cortex in line with previous studies, which indicated that EE might stimulate hippocampal and cortical structural plasticity involving axonal sprouting and synaptogenesis in animals after ischemia.^46^ These results, along with the information obtained from brain connectivity, likely reflected the activated synaptic plasticity and axonal growth in hippocampal and cortical astrocytes may contribute to enhanced synaptic communication following EE.

Recently, the metabolic interaction between astrocytes and neurons has been accepted to serve a critical role in brain functions.^24^ Specifically, astrocytic gap‐junction channels formed by Cx43 provide intercellular communication to neurons.^36^, ^47^ A study reported that inhibited Cx43 level in hippocampus caused cognitive impairments in mice, and elevated level of Cx43 facilitated cognitive function improvement.^48^ Here, we found that stroke significantly decreased the expression of GFAP and Cx43 in the bilateral hippocampus, but EE increased GFAP/Cx43 expression. These results, along with the information obtained from DTI‐based microstructural changes, likely reflected the upregulation of Cx43 expression in the bilateral hippocampal astrocytes may contribute to protected microstructural integrity following EE. It should be noted that intense immunostaining of GFAP‐positive cells associated Cx43 were detected in the ischemic cortex which in agreement with previous studies.^49^ Whereas, ME group showed decreased GFAP/Cx43 immunoreactivity in the peri‐hippocampal cortex. The elevation of Cx43 expression on astrocytes in the ischemic boundary zones has been shown to extend the initial damage via spreading toxic material from infarct to the peri‐infarct tissues.^50^ The data herein indicated that the attenuated Cx43 immunoreactivity in the ischemic region might be beneficial for the brain remodeling following post‐stroke EE intervention.

A striking feature of intense cooperativity between astrocytes and neurons is the energy metabolic interactions through glucose and lactate transporters.^24^ Glucose is the main energy substrate in mammalian brain. Glucose transporters (GLUT1 in astrocytes and GLUT3 in neurons) is crucial to transfer glucose into both neurons and astrocytes.^51^

Monocarboxylate transporters (MCT4 in astrocytes and MCT2 in neurons) transported lactate, one metabolites of glucose, out of astrocytes and into neurons.^52^ Thus, MCTs has been recognized as an important mechanism underlying the metabolic interaction between neuron and astrocyte.^52^

Here, we detected down‐regulation of GLUT1/GLUT3 in the right hippocampus and cortex. In accordance with the change of glucose transporters, significantly decreased expression of MCT4/MCT2 in the corresponding region were measured in pMCAO rats. Evidences have demonstrated that the lack of GLUT1/GLUT3 induced abnormal learning and memory function.^53^, ^54^ In addition, downregulated expression of MCTs in hippocampus may disrupt long‐term memory formation.^24^, ^52^, ^55^ Additionally, reduced level of GLUT1/GLUT3 was measured in the left hippocampus following ischemic stroke. These results showed that disruption of metabolic interactions between neurons and lactate after stroke might induced learning and memory dysfunction. It is noteworthy that cerebral ischemia up‐regulated MCT2/MCT4 in the left hippocampus and cortex, which may be beneficial for the energy supply of brain tissue in ischemic region. However, this compensation was insufficient. PET images provided direct evidence showing obvious reduced energy supply in the ischemic location. Specifically, functional connectivity results based on fMRI and PET imaging indicated that unilateral MCA occlusion caused clearly decreased connectivity in the bilateral hippocampus and peri‐hippocampal cortical regions. EE increased expression of GLUT1, GLUT3, MCT4, and MCT2 in the bilateral hippocampus and right cortex. Several studies demonstrated that up‐regulation of GLUTs and MCTs in the hippocampus or cortex was beneficial to ameliorate memory deficits.^56^, ^57^, ^58^ Of note, EE downregulated MCT4/MCT2 level in the left cortex. Although far from being completely understood, with the information obtained from PET and functional connectivity analysis, we hypothesized that the EE‐induced decrease of the MCTs within the left cortices may play an important role in supplying energy to the left hippocampus. Based on these, the present study involving glucose and lactate transporters provided additional evidences for the brain energy metabolism restorative and learning and memory functions recovery following EE intervention.

The major limitations of this study should be noted. First, the present study was conducted to investigate the EE‐induced plasticity of functional connectivity at 31 days after ischemic stroke. The dynamic changes of connectivity between hippocampus and peri‐hippocampal cortical regions after EE need to be further studied. In addition, very little is known about the correlation between the results of fMRI and PET. Further study designed to investigate the relationship between the EE‐induced changes of functional activity and glucose metabolism following stroke is very necessary. Although future studies are needed, our findings provide evidence that the three‐phase EE paradigm might be an ideal post‐stroke rehabilitation program for improving hippocampal–cortical connectivity plasticity and ameliorated post‐stroke memory deficits following ischemic stroke.

## AUTHOR CONTRIBUTIONS

Yun Lu: Conceptualization, Methodology, Investigation, Writing ‐ original draft. Mingcong Li: Methodology, Investigation. Yuming Zhuang: Methodology, Investigation. Ziyue Lin: Methodology, Investigation, Data curation. Binbin Nie: Methodology, Investigation, Software. Jianfeng Lei: Methodology, Investigation, Software. Yuanyuan Zhao: Methodology, Investigation, Software. Hui Zhao: Conceptualization, Supervision, Writing ‐ review & editing.

## FUNDING INFORMATION

This work was supported by the National Natural Science Foundation of China (82174471). We appreciate Zhongxin Xiao for his help in microscope imaging of GFAP and Cx43 expression in the Figure 7.

## CONFLICT OF INTEREST STATEMENT

The authors declare no competing financial interests.

## Supporting information


Appendix S1

## Data Availability

The data that support the findings of this study are available from the corresponding author upon reasonable request.
