# Supplementary material for: Combination of fMRI and PET reveals the beneficial effect of three‐phase enriched environment on post‐stroke memory deficits by enhancing plasticity of brain connectivity between hippocampus and peri‐hippocampal cortex
Source: CNS Neurosci Ther. 2023 Sep 27;30(3):e14466. doi: 10.1111/cns.14466 (PMC10916434; doi:10.1111/cns.14466)
Supplement: Supplementary file 1 — Appendix S1 [file CNS-30-e14466-s001.zip › cns14466-sup-0001-AppendixS1.docx]

**Supplementary Information**

Table S1 Difference of FC with the right hippocampus as the seed.

|  | Cluster level | | Coordinate [mm] | | | Region |
| --- | --- | --- | --- | --- | --- | --- |
|  | *K*_E_ | *T* | x | y | z |  |
| MS < SS | 76 | 4.03 | -3.3 | 6.1 | -7.1 | Hip_L |
|  | 42 | 3.90 | -5.8 | 4.0 | -7.1 | Ent_L |
|  | 59 | 4.97 | -0.2 | 2.0 | -2.3 | RSC_L, RSC_R |
| ME > MS | 205 | 4.94 | -4.0 | 2.0 | -8.5 | Ent_L, Hip_L, RSC_L |

Table S2 Difference of FC with the left hippocampus as the seed.

|  | Cluster level | | Coordinate [mm] | | | Region |
| --- | --- | --- | --- | --- | --- | --- |
|  | *K*_E_ | *T* | x | y | z |  |
| MS < SS | 70 | 5.40 | 6.5 | 3.2 | -8.5 | Ent_R |
|  | 44 | 3.84 | -0.4 | 1.4 | -3.2 | RSC_L, RSC_R |
| ME > MS | 126 | 4.23 | 0.6 | 1.8 | -6.1 | RSC_R |
|  | 68 | 4.75 | 7.1 | 6.4 | -3.7 | Ent_R |
|  | 61 | 4.49 | 5.4 | 6.7 | -7.1 | Ent_R, Hip_R |

Table S3 Significant differences in metabolic connectivity between regions.

| MS *vs.* SS | | |  | ME *vs.* MS | | |
| --- | --- | --- | --- | --- | --- | --- |
| Brain region | Brain region | *P* values |  | Brain region | Brain region | *P* values |
| Hip_L | Hip_R | 0.013 |  | Hip_L | Ent_R | 0.044 |
| Hip_L | DG_R | 0.002 |  | Hip_L | RSC_R | 0.008 |
| Hip_L | Ent_R | 0.001 |  | DG_L | Ent_R | 0.016 |
| Hip_L | RSC_R | < 0.001 |  | DG_L | Cg_R | 0.042 |
| DG_L | Hip_R | < 0.001 |  | DG_L | RSC_R | 0.014 |
| DG_L | DG_R | 0.006 |  |  |  |  |
| DG_L | Ent_R | < 0.001 |  |  |  |  |
| DG_L | Cg_R | 0.037 |  |  |  |  |
| DG_L | RSC_R | 0.003 |  |  |  |  |
| Ent_L | DG_R | 0.006 |  |  |  |  |
| Cg_L | DG_R | 0.001 |  |  |  |  |
| RSC_L | Hip_R | 0.036 |  |  |  |  |
| RSC_L | DG_R | 0.018 |  |  |  |  |

Table S4 Global efficiency of each group.

| Group | Global efficiency |
| --- | --- |
| SS | 0.702 |
| SE | 0.877 |
| MS | 0.215 |
| ME | 0.488 |

Table S5 Intergroup differences of global efficiency.

|  | *P* values |
| --- | --- |
| MS *vs.* SS | < 0.0001 |
| ME *vs.* MS | 0.0209 |

Table S6 Sequence parameters for acquisition/reconstruction, spatial resolution and duration.

| Imaging | Sequence | Parameters |
| --- | --- | --- |
| T2WI | a fast spin-echo pulse sequence | TR = 4400 ms, TE = 45 ms, Matrix size = 256×256, flip angle = 180°, Field of View = 3.3×3.3 cm, 38 slices, slice thickness = 0.7 mm, slice gap = 0, Scan time = 4 min. |
| T2 mapping | a multi-slicemulti-echo (MSME) sequences | 16 TEs, from 11 to 176 ms, TR = 2500 ms, flip angle = 180°, FOV = 3.3×3.3 cm, Matrix size = 256 × 256, 5 slices, slice thickness = 1 mm, Scan time = 8 min. |
| DTI | an axial single-shot spin echo-planar imaging sequence | TR/TE = 6300/25 ms, 30 diffusion encoding directions, two b values = 0 and 1000 s/mm2, Matrix size = 128×128, Flip angle = 90°, Field of View = 3.5×3.5 cm2, Number of excitation = 1, Scan time = 10 min. |
| BOLD | an EPI-SE-FOVsat sequence | TR = 2000 ms, TE = 11 ms, Matrix size = 80×64, flip angle = 90°, Field of View = 2.5×2.0 cm, 38 slices, slice thickness = 0.7 mm, slice gap = 0, Scan time = 8 min. |
| PET/CT |  | The scanning procedure consisted of a 20-min static acquisition period and an 8-min attenuation-correction CT scan. |


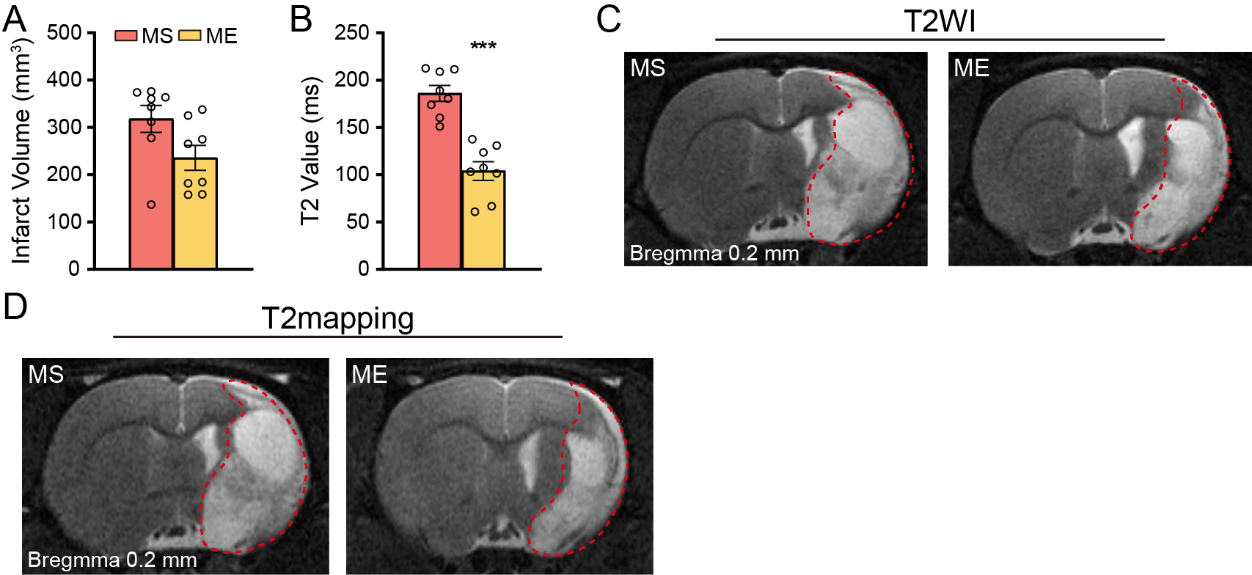


Fig.S1 The difference of infarct volume between MS and ME groups. (A) Quantitative analysis of infarct volume. (B) Quantitative analysis of T2 value in infarct region. Anatomical distribution of infarct areas overlaid in (C) T2WI and (D) T2mapping image. Red dotted line represented the infarct areas. Data were present as mean ± SEM, N = 8 rats per group.
