# Supplementary material for: Combination of fMRI and PET reveals the beneficial effect of three‐phase enriched environment on post‐stroke memory deficits by enhancing plasticity of brain connectivity between hippocampus and peri‐hippocampal cortex
Source: CNS Neurosci Ther. 2023 Sep 27;30(3):e14466. doi: 10.1111/cns.14466 (PMC10916434; doi:10.1111/cns.14466)

Full unedited blot for Figure 6A

Blot for SYN in right hippocampus	Blot for GAPDH in right hippocampus
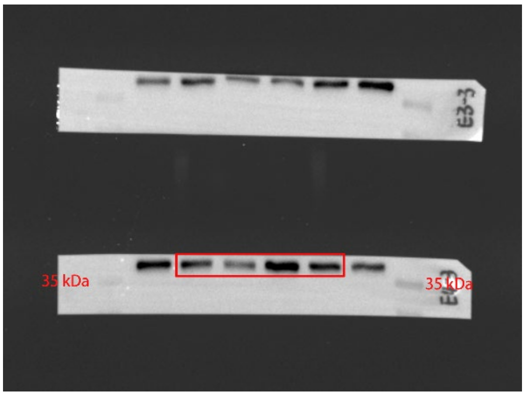	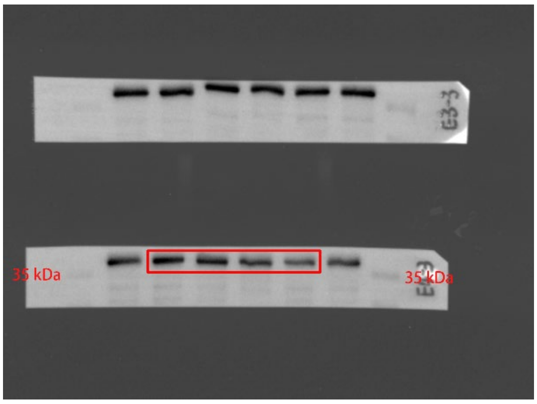
Blot for SYN in left hippocampus	Blot for GAPDH in left hippocampus
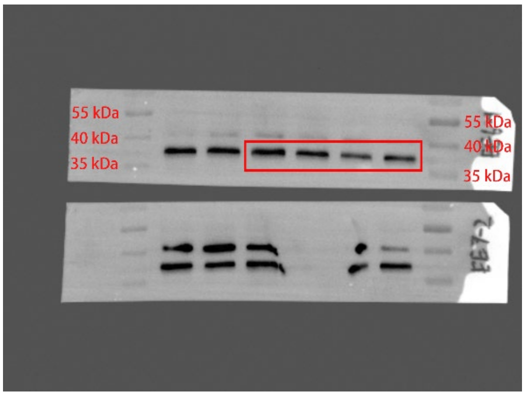	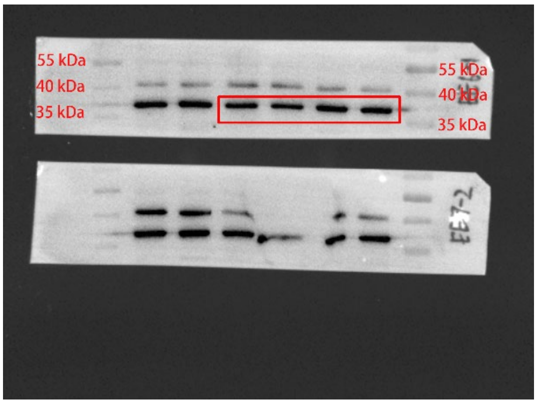
Blot for SYN in right cortex	Blot for GAPDH in right cortex
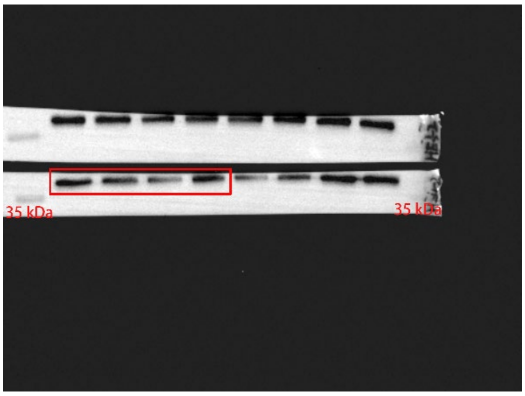	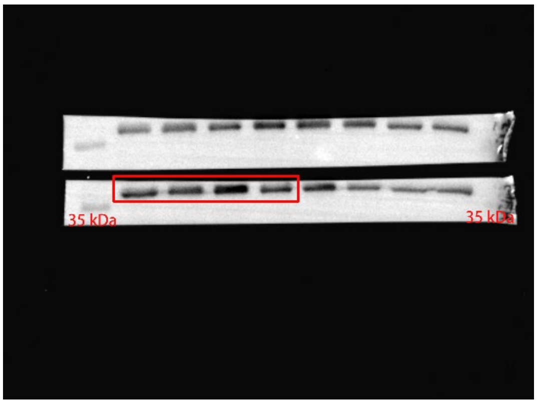
Blot for SYN in left cortex	Blot for GAPDH in left cortex

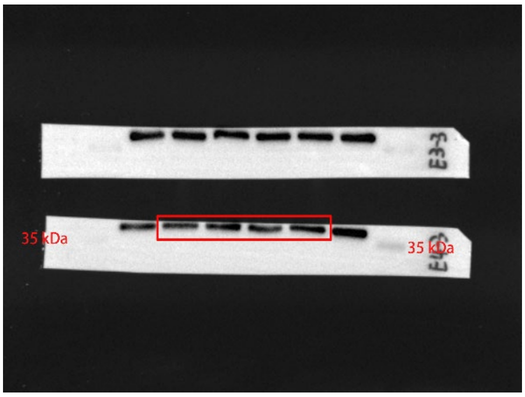

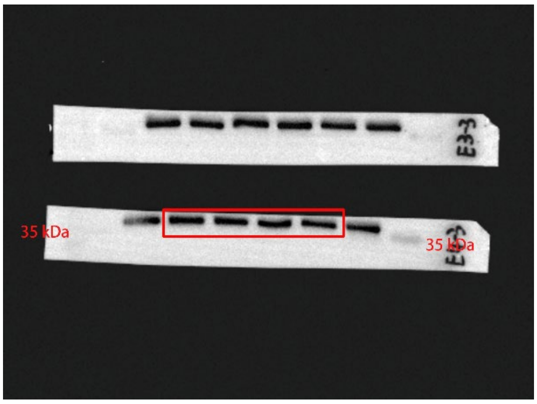

Full unedited blot for Figure 6B

Blot for GAP-43 in right hippocampus

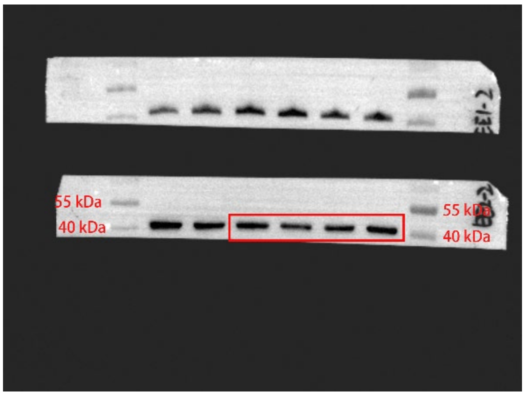

Blot for GAPDH in right hippocampus

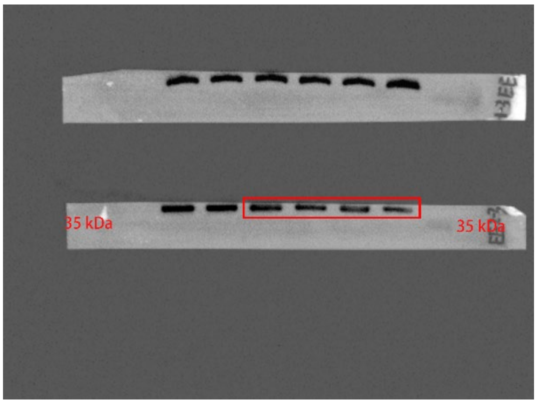

Blot for GAP-43 in left hippocampus

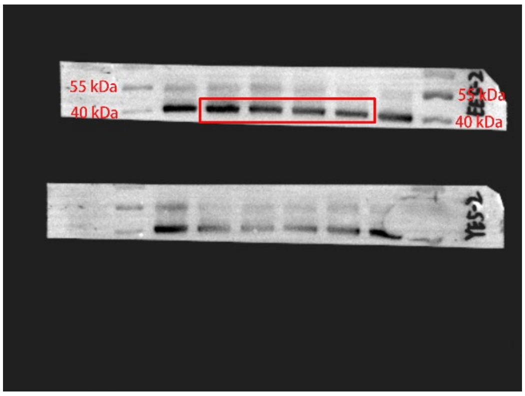

Blot for GAPDH in left hippocampus

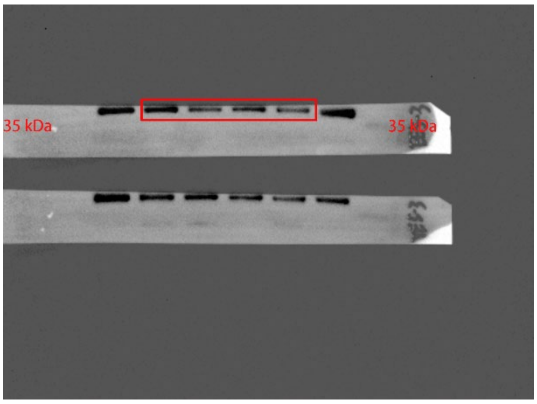

Blot for GAP-43 in right cortex

Blot for GAPDH in right cortex

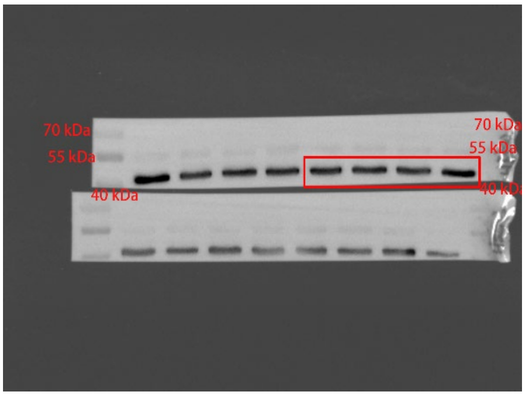

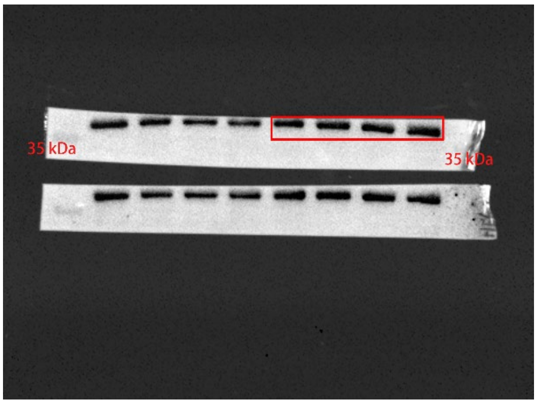

Blot for GAP-43 in left cortex

Blot for GAPDH in left cortex

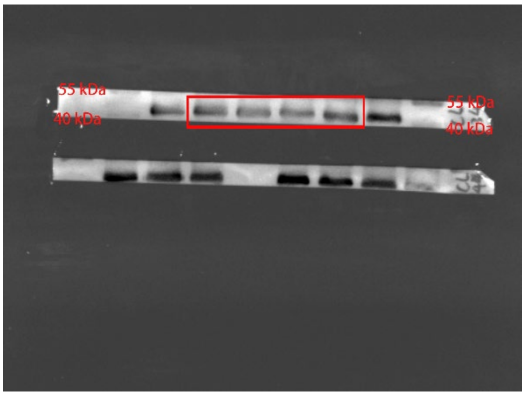

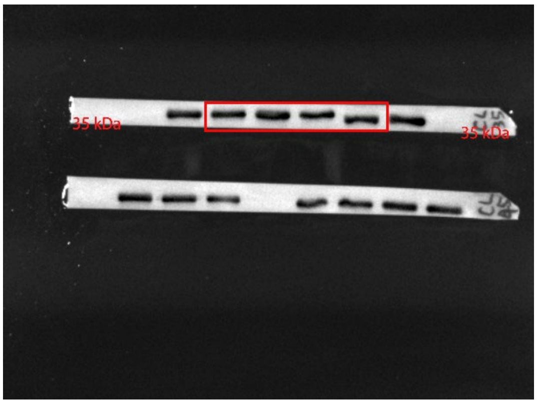

Full unedited blot for Figure 8A

Blot for GLUT1 in right hippocampus

Blot for GAPDH in right hippocampus

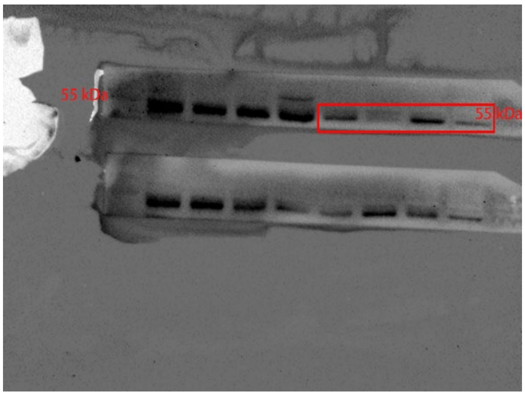

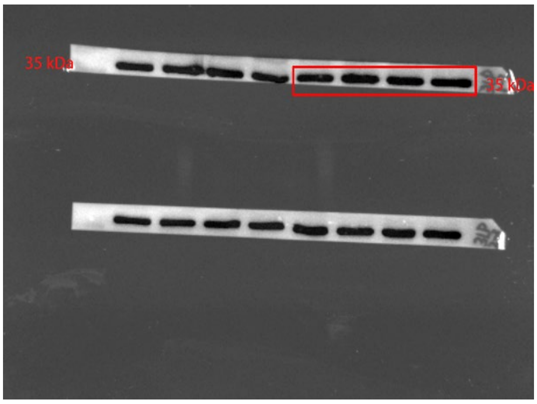

Blot for GLUT1 in left hippocampus

Blot for GAPDH in left hippocampus

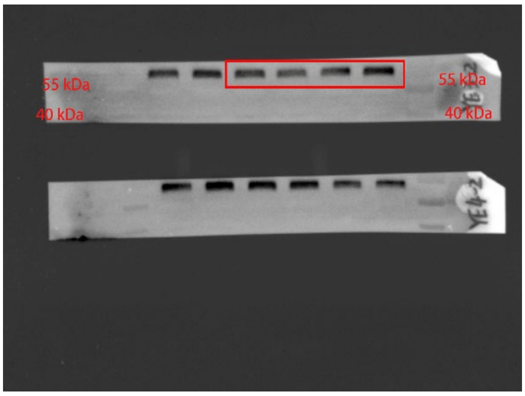

Blot for GLUT1 in right cortex

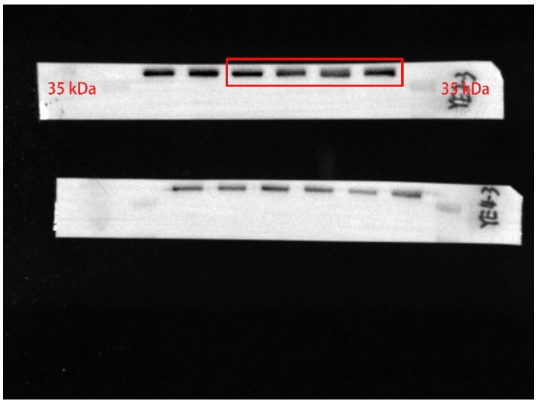

Blot for GAPDH in right cortex

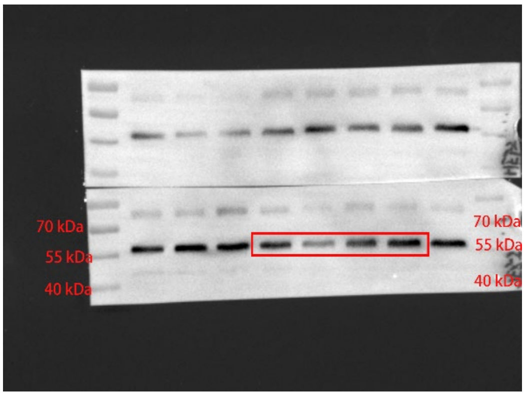

Blot for GLUT1 in left cortex

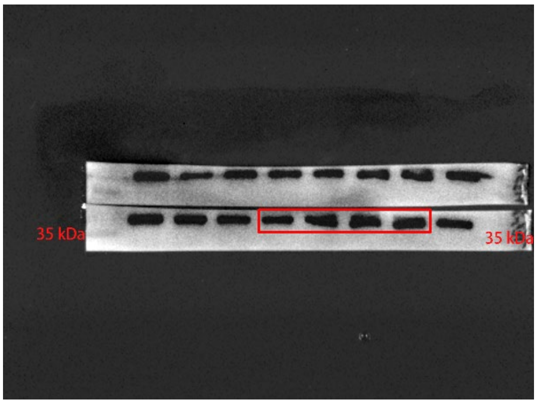

Blot for GAPDH in left cortex

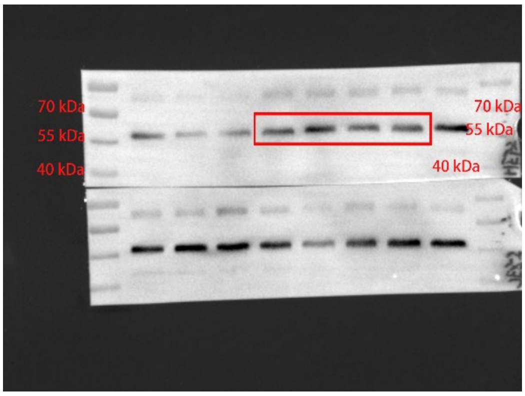

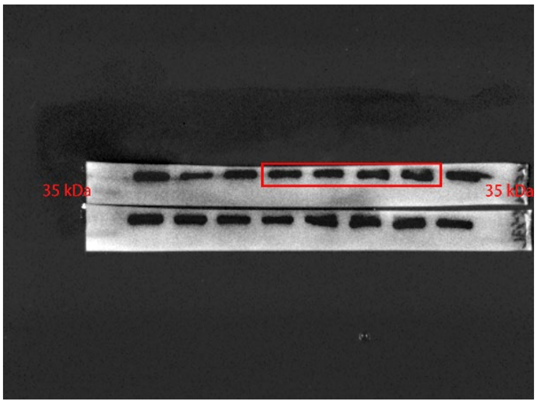

Full unedited blot for Figure 8B

Blot for GLUT3 in right hippocampus	Blot for GAPDH in right hippocampus

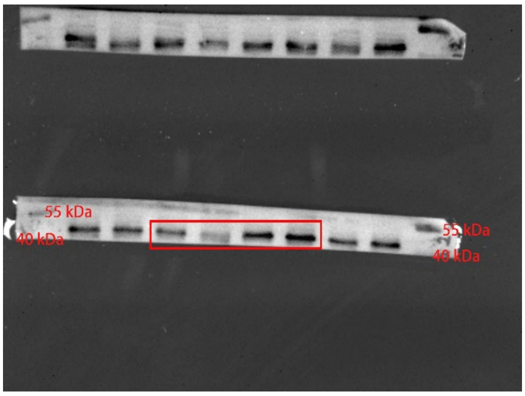

Blot for GLUT3 in left hippocampus

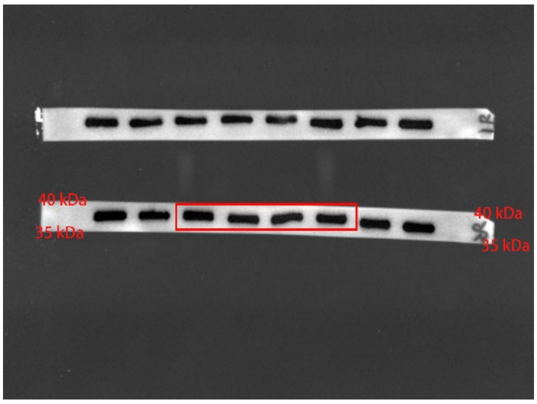

Blot for GAPDH in left hippocampus

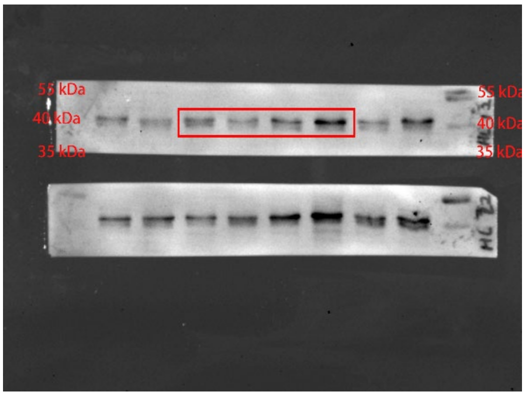

Blot for GLUT3 in right cortex

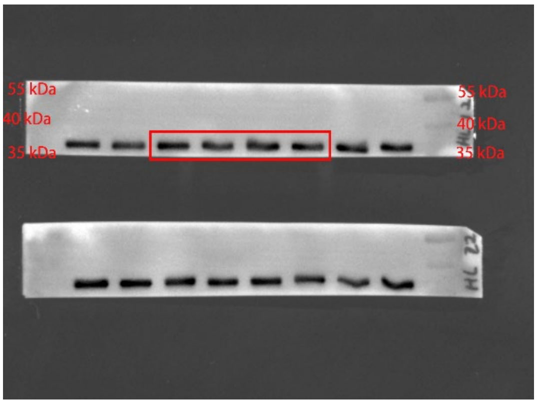

Blot for GAPDH in right cortex

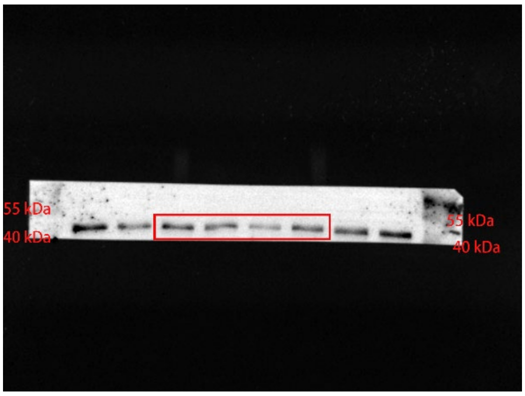

Blot for GLUT3 in left cortex

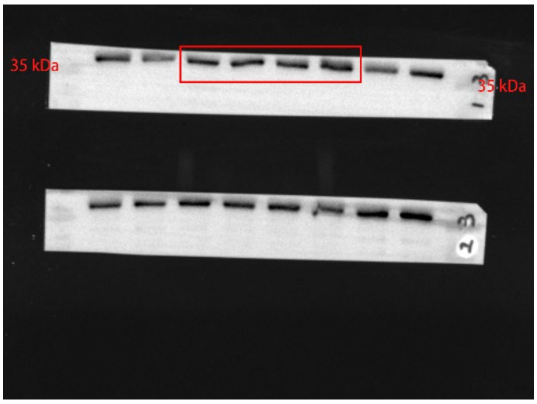

Blot for GAPDH in left cortex

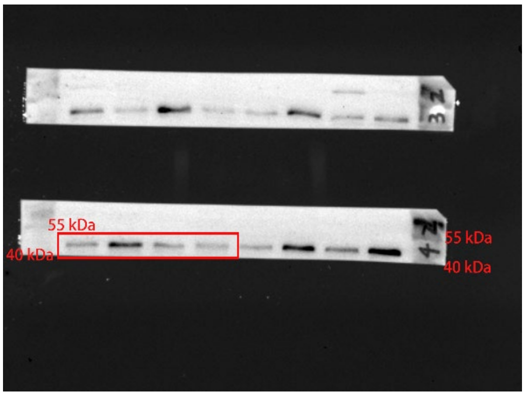

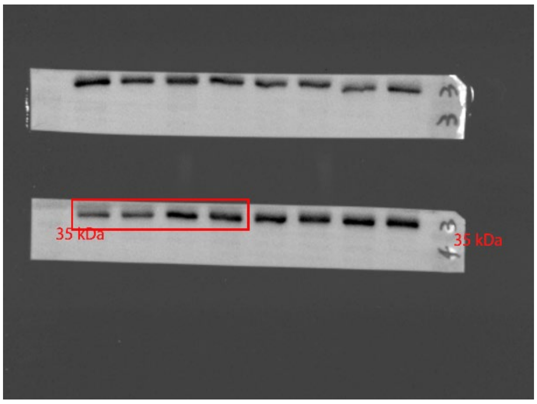

Full unedited blot for Figure 8C

Blot for MCT4 in right hippocampus	Blot for GAPDH in right hippocampus
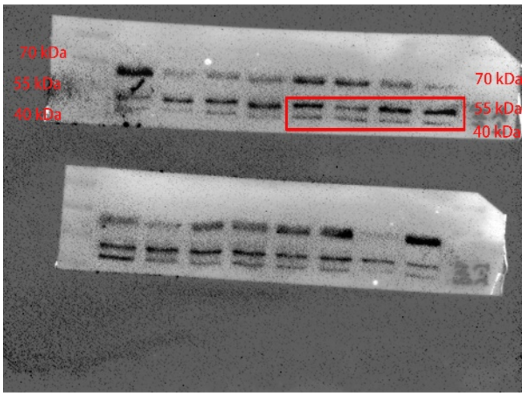	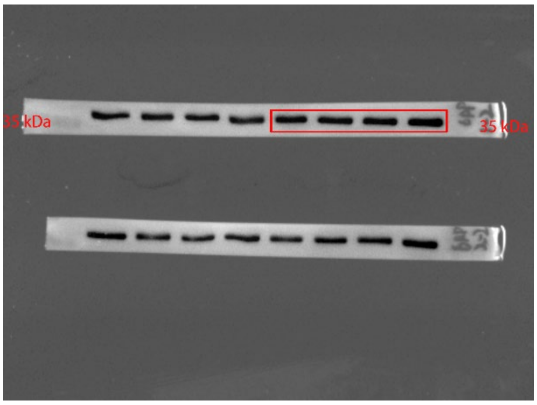
Blot for MCT4 in left hippocampus	Blot for GAPDH in left hippocampus
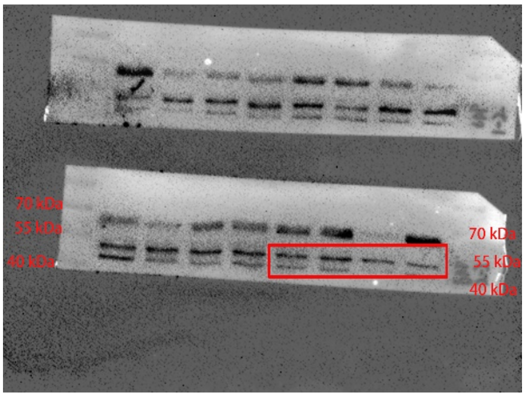	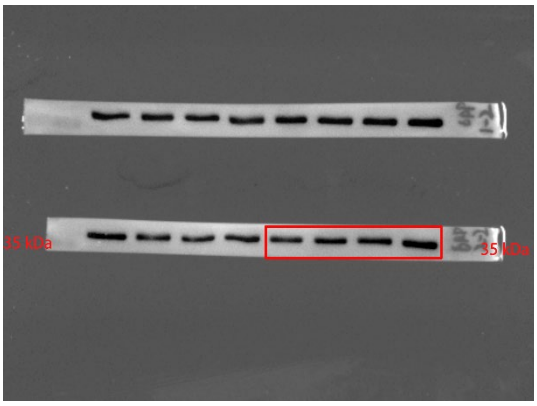
Blot for MCT4 in right cortex	Blot for GAPDH in right cortex
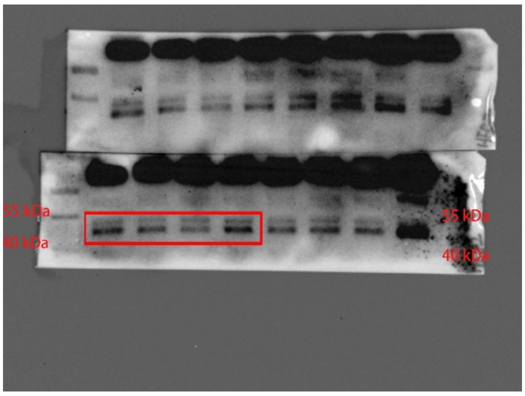	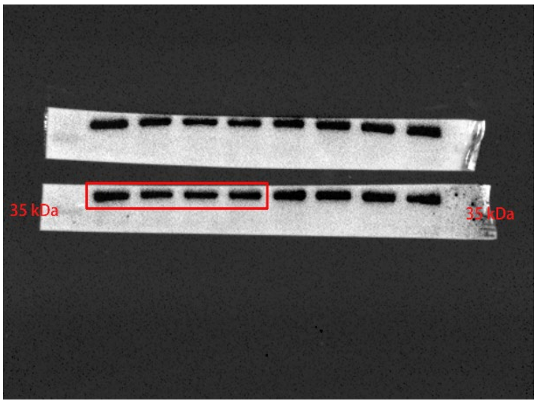
Blot for MCT4 in left cortex	Blot for GAPDH in left cortex

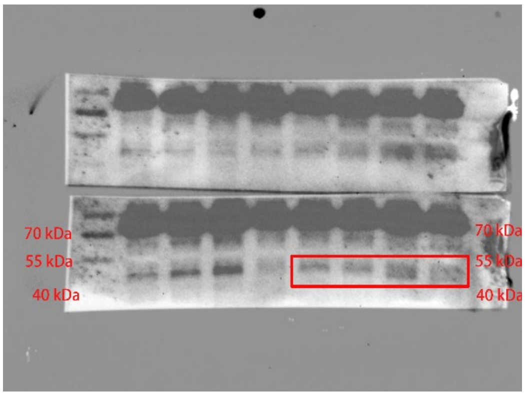	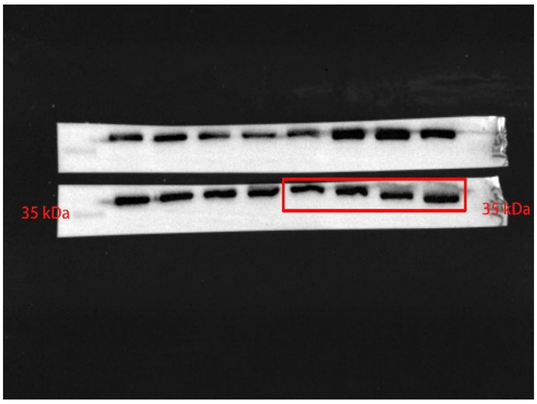

Full unedited blot for Figure 8D

Blot for MCT2 in right hippocampus	Blot for GAPDH in right hippocampus
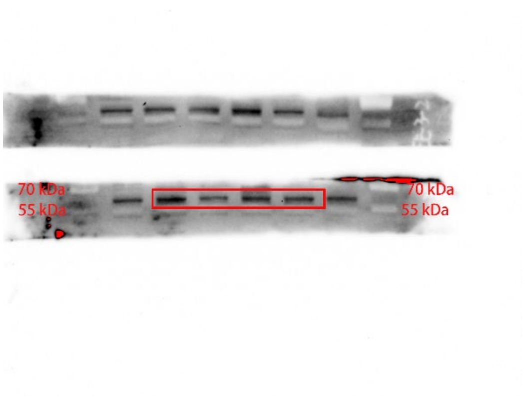	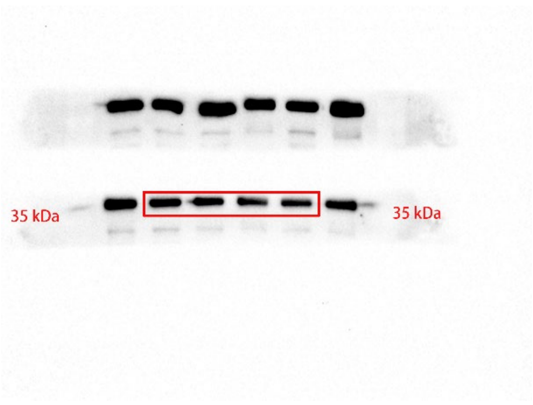
Blot for MCT2 in left hippocampus	Blot for GAPDH in left hippocampus
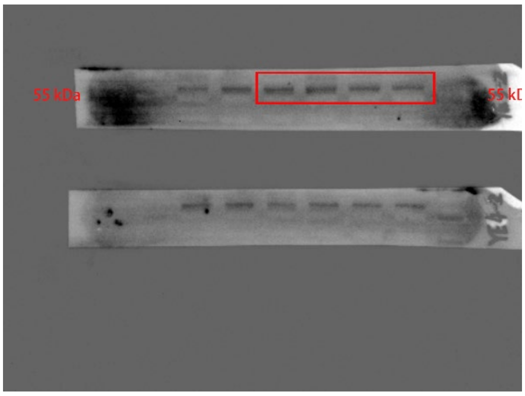	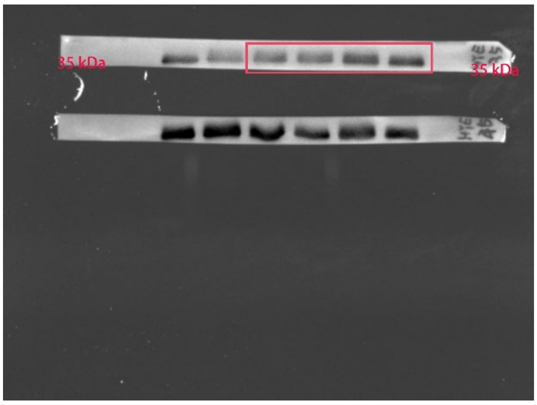
Blot for MCT2 in right cortex	Blot for GAPDH in right cortex

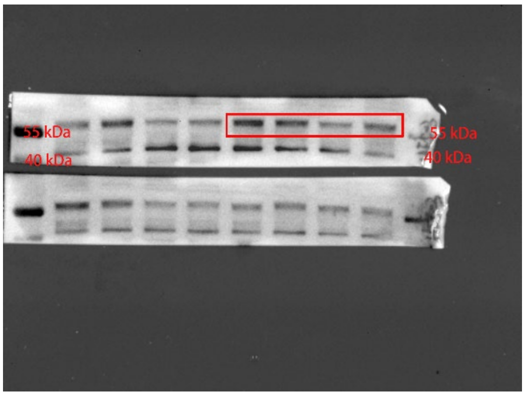

Blot for MCT2 in left cortex

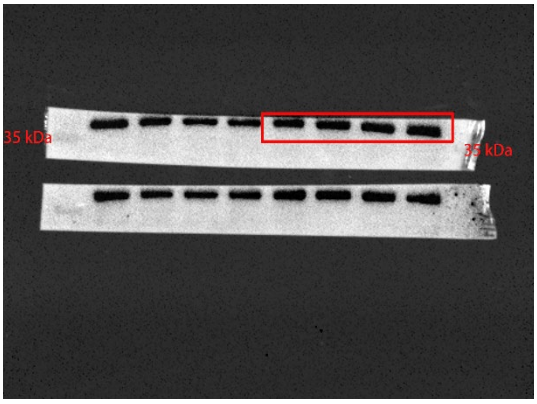

Blot for GAPDH in left cortex

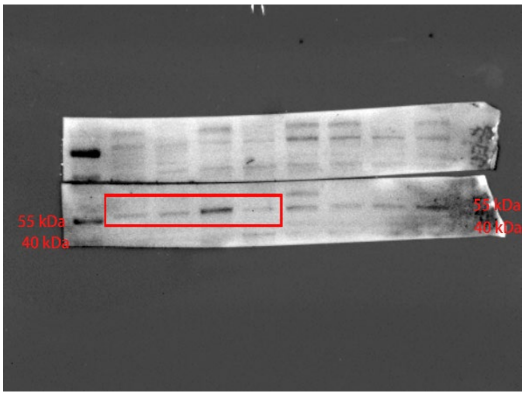

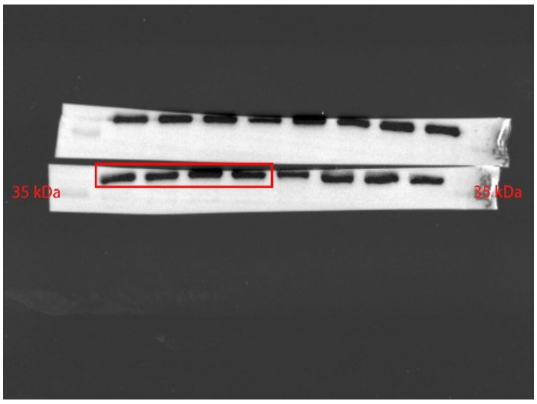

Supplement: Supplementary file 1 — Appendix S1 [file CNS-30-e14466-s001.zip › cns14466-sup-0002-AppendixS2.pdf]
